# Elevation Matters More than Season in Shaping the Heterogeneity of Soil and Root Associated Ectomycorrhizal Fungal Community

**DOI:** 10.1128/spectrum.01950-21

**Published:** 2022-01-12

**Authors:** Sai Gong, Bang Feng, Si-Peng Jian, Geng Shen Wang, Zai-Wei Ge, Zhu Liang Yang

**Affiliations:** a CAS Key Laboratory for Plant Diversity and Biogeography of East Asia, Kunming Institute of Botany, Chinese Academy of Sciences, Kunming, Yunnan, China; b Yunnan Key Laboratory for Fungal Diversity and Green Development, Kunming Institute of Botany, Chinese Academy of Sciences, Kunming, Yunnan, China; c University of Chinese Academy of Sciences, Beijing, China; Nanyang Technological University

**Keywords:** ectomycorrhizal fungi, altitude, season, spatiotemporal variation, Baima Snow Mountain

## Abstract

Ectomycorrhizal (EcM) fungi play important roles in forest ecosystems, and their richness and composition can change along with elevation and season changes. However, no study has estimated the relative importance of altitudinal and seasonal heterogeneity in predicting the distribution of EcM fungal communities by simultaneously considering different sample types (root versus soil). In this study, we collected root and soil samples along a > 1,500-m elevation gradient during wet and dry seasons from Baima Snow Mountain, located in “the Mountains of Southwest China,” one of the 34 biodiversity hot spots, and we analyzed them using next-generation sequencing. Regardless of the sample type, similar EcM fungal richness pattern with increasing elevation (decline in the forest zone, and an increase at the alpine meadow zone) and strong community turnovers among different elevational zones and between two seasons were detected, and changes of EcM fungal community similarity on 400-m altitude gradient were equivalent to the community turnover between dry and wet seasons. Elevation and edaphic factors were shown to have the largest effects on EcM fungal community. The heterogeneity of richness and community composition was stronger among different elevational zones than across different seasons, mainly because the elevation variations in the EcM fungal community were shaped by the combined effects of different environmental factors, while seasonal changes were mainly controlled by temperature and fast-changing soil nutrients.

**IMPORTANCE** Altitude and season represent two important environmental gradients that shape the structure of biome, including the heterogeneity of EcM fungi. Previous studies have separately considered the influences of altitude and season on EcM fungal communities, but the relative importance of altitude and season is still unknown. The present study revealed that elevation influences the heterogeneity of EcM fungal community more than season; this may be because the variability of environmental factors is higher across different elevations than that across seasons.

## INTRODUCTION

The decline in soil biodiversity is one of the main reasons for the impact of environmental changes on forest processes (1). As a major guild of soil biome, ectomycorrhizal (EcM) fungi play an important role in nutrient cycling via exchanging soil nutrients with host plants for photosynthetic carbon (2). Furthermore, they can provide substantial protection to the host plants against soilborne pathogens by ensheathing the root tips and acidifying the soil (3). Studies have reported that the decline of EcM fungal diversity caused by increasing atmospheric temperature (4, 5) and long-term nitrogen (N) deposition (6, 7) will not be conducive to the establishment of their host plant populations. Under drought stress (low soil moisture), the EcM fungal community structure can change to help maintain the host N supply (8–10). Hence, an increasing number of ecological studies are being focused on EcM fungi. In the past decade, with rapid advancements in DNA sequencing technology, the diversity of EcM fungi has been studied on global (11), regional (1, 12, 13), and local (14–17) scales. Many of these studies have focused on elucidating the underlying mechanism shaping the spatiotemporal distribution pattern of EcM fungal richness and community composition (15, 18–20).

Elevation and season are two comprehensive environmental gradients that have been widely studied to understand the dynamics of soil microbial community in space and time. Elevation gradient usually leads to dramatic changes in several biotic and abiotic factors, such as temperature and type of vegetation, within a relatively narrow geographical range (20–22), whereas seasonal changes usually cause alternating climatic conditions and affect nutrient transfer by changing the plant physiology. For example, winter season is associated with low temperature and large input of plant residues (e.g., litter and dead roots) to soil (23, 24). Such environmental heterogeneities across different elevations and seasons remarkably influence the spatiotemporal dynamics of soil biomes. For example, changes in temperature and moisture are two important factors that affect the spatiotemporal composition of soil fungi by directly controlling the enzymatic production and activities of fungi (25) and by indirectly affecting the plant community and soil nutrients (26, 27). In addition, the variation of vegetation cover type (12); soil pH, organic matter (OM), N, phosphorus (P), and potassium (K) content (28, 29) along altitude gradient; and the variation of litter inputs and the underground allocation of photosynthate between seasons (30) can also shape the fungal community structure. This holds true for EcM fungi, and various spatial, host-related, climatic, and soil factors—or their combinations—are key explanatory factors that determine the changes in EcM fungal community along different environmental gradients (20, 31, 32). For example, the fine-scale changes in soil properties, such as its pH and total nitrogen (TN) content, can explain the variability in EcM fungal communities in alpine habitats (33). Similarly, fluctuations in environmental factors, such as temperature, humidity, and available nutrients, can also lead to significant seasonal variations in EcM fungal communities (34–38).

When the effect of elevation and season are considered together, the degree of variation of environmental factors along a large altitude gradient (> 700 m) is often greater than that between different seasons; this can, in turn, result in the stronger influence of altitude on the distribution of soil bacterial and fungal communities at local scale than season (24, 39). Particularly, slow-changing soil properties—pH, total carbon, N, P, and K—across large spatial scales (40–42) are strong predictors of the spatial distribution of microbial communities. In contrast, fast-changing environmental covariates, such as climate variables, soil moisture, and available nutrient, are likely the main factors that drive the seasonal variation in the composition of soil microbial communities (41). The dynamics of EcM fungi, like other guilds of soil microbiomes, are influenced jointly by different environmental factors. Hence, we hypothesized that an elevational gradient of > 700 m would lead to high heterogeneity in environmental variables, thereby resulting in high heterogeneity of EcM fungi compared to that found between different seasons. Furthermore, EcM fungi tightly rely on both the host (biotic component) and the surrounding environment (abiotic component) for resource acquisition via well-differentiated symbiotic compartments (i.e., mantle and Hartig net in roots, and the external mycelium spread in soil) (28). Therefore, we also hypothesized that the EcM fungal communities from root and soil are inconsistent in response to altitude and seasonal changes.

The area of the “mountains of Southwest China” is one of the 34 biodiversity hot spots of the world (43). Studies on fruiting bodies have shown that it is one of the most biodiverse centers of EcM mushrooms (44–48). However, only sporadic studies have been conducted on the soil- or root-associated EcM fungal richness and community composition of this region (49, 50). The Baima Snow Mountain National Nature Reserve, located in this biodiversity hot spot, has a large altitude span (2,040 m to 5,429 m) and distinct dry (from December to May next year) and wet (from June to November) seasons; thus, it provides favorable conditions for studying the community variations in EcM fungi between the two seasons along an altitude gradient. In this study, we sampled root and bulk soil samples across an altitude gradient of >1,500 m during wet and dry seasons and used next-generation sequencing to study the EcM fungal richness and community composition in the Baima Snow Mountain. We aimed to: (a) assess the diversity of EcM fungi in both forest and alpine meadow zones, (b) reveal the pattern of spatiotemporal dynamics of EcM fungal richness and community compositions, and (c) quantify the relative importance of different factors—elevation, season, spatial, host, climate, and soil—on shaping the observed spatiotemporal dynamics of EcM fungi.

## RESULTS

### Variations in environmental variables across different elevations and seasons.

**FIG 1:**
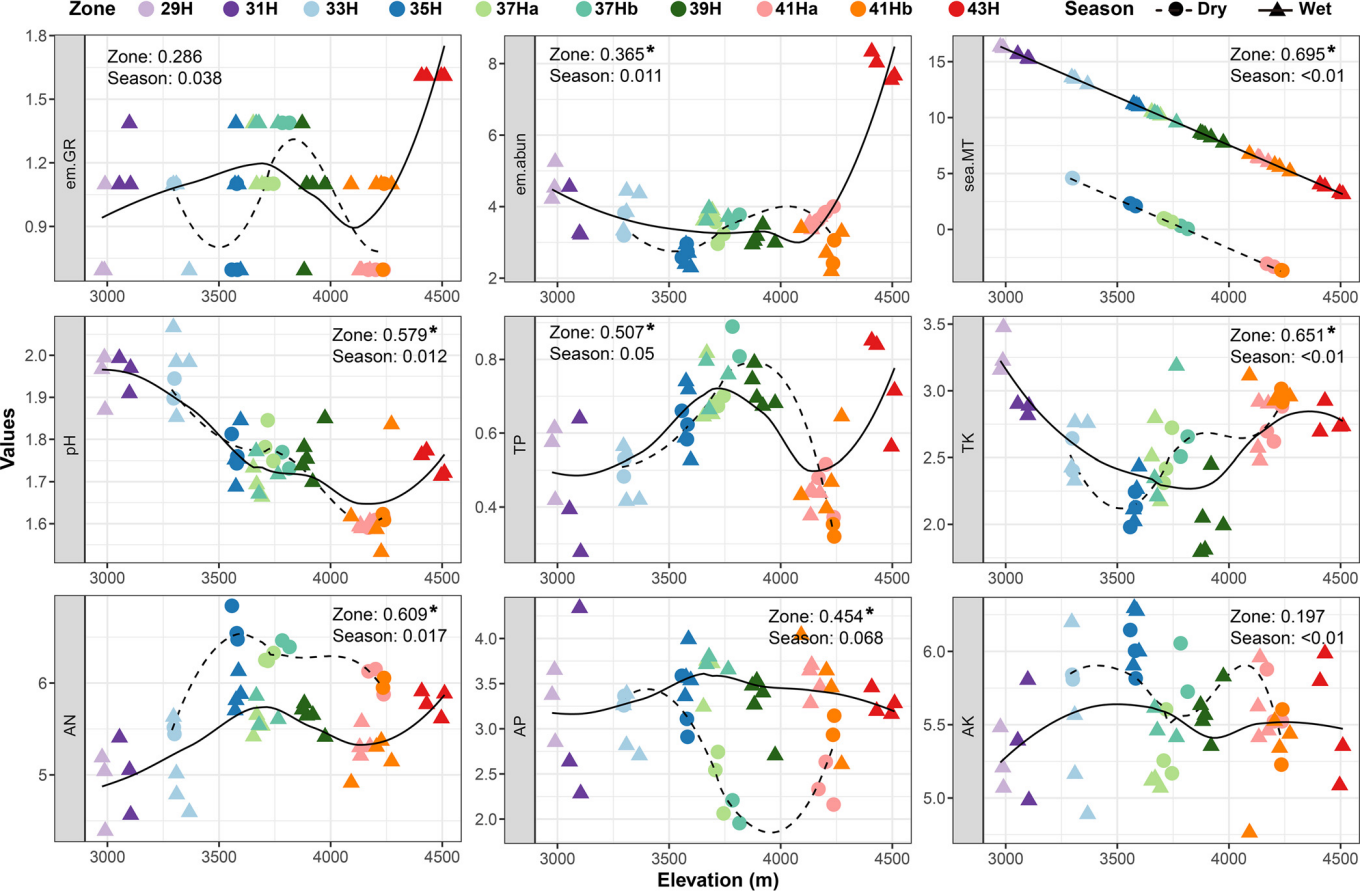
Changes in environmental variables along the altitude gradient and between wet and dry seasons. Circles and dash lines represent dry season, whereas triangles and solid lines represent wet season. The fitted lines were obtained using the non-parametric smoother LOESS. Numbers after “Zone” and “Season” are the R^2^ values and represent the variation in variables that can be explained by altitude or season detected using two-way analysis of variance. *, the significant level at *P < *0.05; em.GR, richness of EcM plant at the genus level; em.abun, the number of individuals of each EcM genus; sea.MT, dry-season and wet-season mean temperature; pH, soil acidity and alkalinity; TP, total phosphorus; TK, total potassium; AN, alkaline-hydrolysable nitrogen; AP, available phosphorus; AK, available potassium. Log transformation for em.GR, em.abun, pH, TP, TK, AN, AP, and AK.

Detected environmental variables, except for richness of EcM plants at the genus level (em.GR) and available potassium (AK), varied significantly with robust R^2^ values along the elevation gradient, whereas only available phosphorus (AP) varied between the two seasons at the margin of significance (*P = *0.084) (Fig. 1).

### General characteristics of EcM fungal communities.

In total, 1,365,440 reads were assigned to 3,416 EcM fungal amplicon sequence variants (ASVs) (691 operational taxonomic units [OTUs]), representing two phyla (Basidiomycota and Ascomycota), 39 phylogenetic lineages, and 66 genera. Taxa within Basidiomycota showed a significant dominance based on either the detected EcM fungal genera (52 in Basidiomycota vs 14 in Ascomycota) or the top 10 genera with a high relative abundance (nine in Basidiomycota vs one in Ascomycota) (Table S2). Rarefaction curves of observed richness did not reach an asymptote for any sample type (Fig. S3a). However, the estimated richness (Chao) included 3,932 ASVs, which was not far from the observed richness (Fig. S3b).

### Variations in the EcM fungal community across elevations, seasons, and host genera.

**FIG 2:**
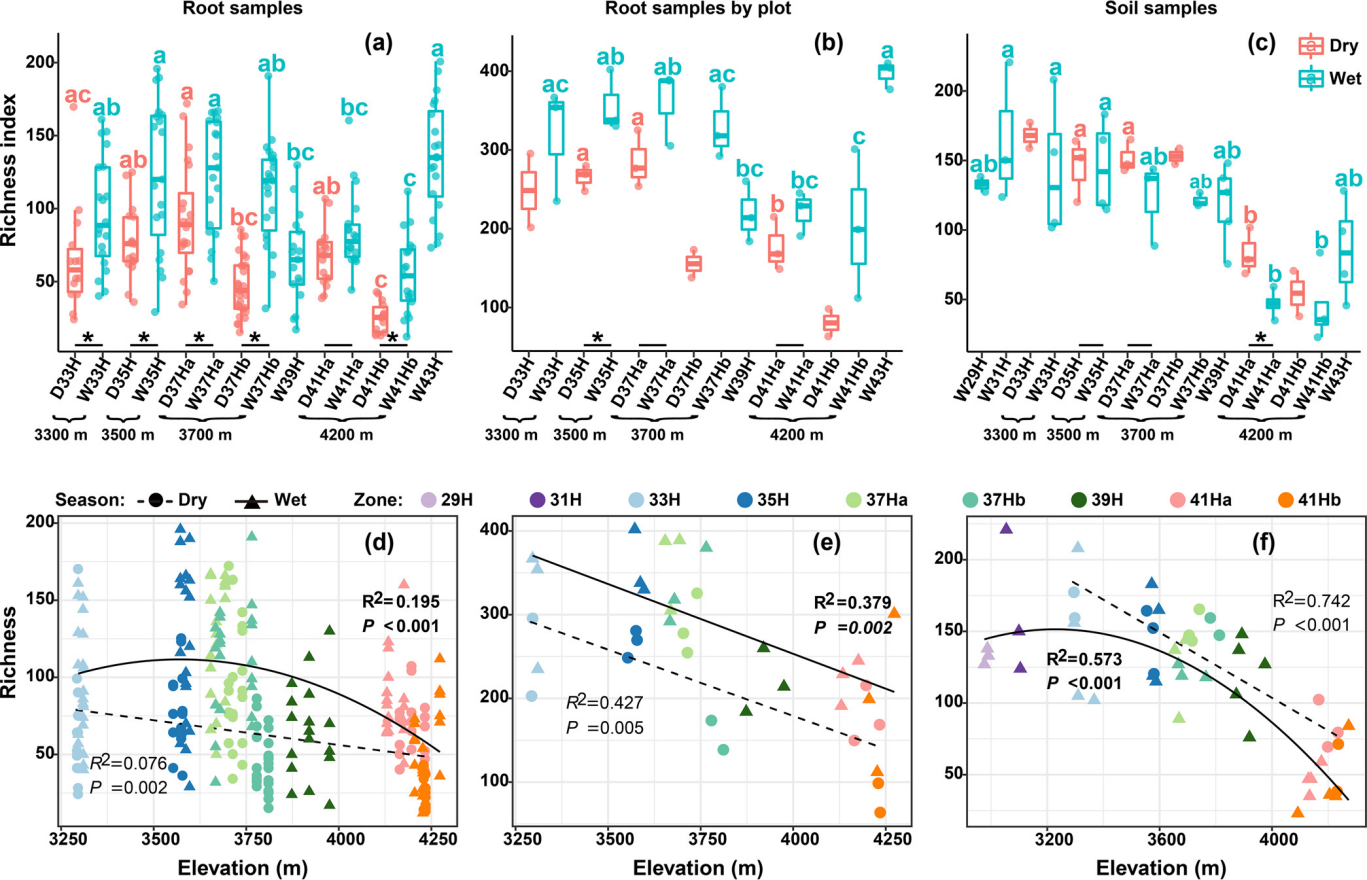
Elevation and seasonal variations in EcM fungal richness. The left, middle, and right columns of graphs are based on single root samples, root samples merged via plots, and soil samples, respectively. In the graphs a to c, the distribution of data is illustrated using box and whisker plots; whiskers represent the range between the minimum and maximum observed values, boxes represent the first and third quartile, horizontal lines denote the median, and outlier observations are plotted as individual points. The red and green boxes correspond to dry and wet seasons, respectively. Different lowercase letters on the top of boxes indicate significant differences among elevation zones at *P* < 0.05. The short solid line represents the ANOVA analysis between dry and wet seasons in a same elevation. Asterisks indicate significant difference (*P < *0.05) between seasons. Zones without letters on the top of boxes or short solid lines below boxes had number of samples that did not meet the criteria (*n* > 3) of statistical test; they were not included in the statistical analysis. In the graphs d to f, the regression lines show the richness patterns along the altitude gradient in forest zone. Circles and dash lines represent dry season; triangles, solid lines, and bold R^2^ and *p* represent wet season.

The EcM fungal richness in the forest zone showed an overall monotonically decreasing pattern with increasing altitude (Fig. 2). However, different seasonal patterns of EcM fungal richness were observed for the root and soil samples. For the root samples, the richness was high in the wet season (Fig. 2a, b, d, and e), whereas for the soil samples, the richness was high in the dry season (Fig. 2c and f). In addition, the alpine meadow showed a significant increase in richness when compared with its adjacent subalpine forest (Fig. 2a to c). The Chao1 and Shannon indices showed an overall similar pattern with the observed richness (Fig. S4).

**FIG 3:**
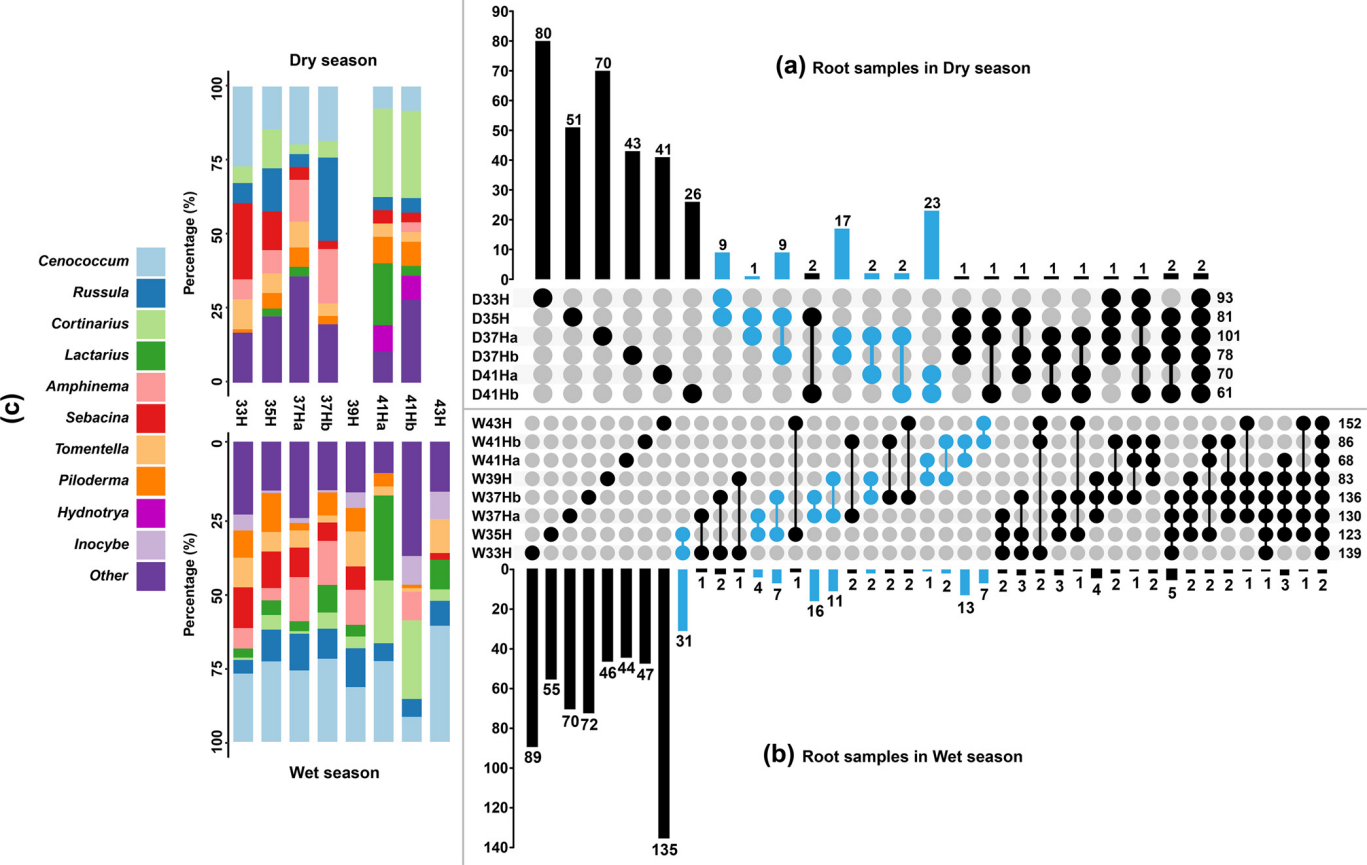
UpSetView plots (a to b) and tableau stacked bar charts (c) based on EcM fungal ASVs (relative abundance > 0.1%) from root samples. The plots show the number of shared and exclusive ASVs among zones and the relative number of ASVs in each genus. The percentage in stacked bar chart is the ratio of the numbers of ASVs in each genus at each sampling zone to the total number of ASVs at that zone. The dot matrix and the corresponding bar above it represent the numbers of shared and exclusive ASVs among zones. The adjacent zones are highlighted in sky blue.

Strong turnovers of EcM fungal community compositions were observed across different elevations and seasons. For root and soil samples in dry (Fig. 3a, Fig. S5a) and wet seasons (Fig. 3b, Fig. S5b), a total of 387, 702, 430, and 655 EcM fungal ASVs with more than 0.1% relative abundance were generated, and approximatively 80.36%, 79.49%, 83.95%, and 68.24% of the total ASVs, respectively, were exclusively found in a single elevation zone. Meanwhile, nearly half of the ASVs were exclusively found only in the dry or wet season (Fig. S3c, d). *Cenococcum* spp. (ASV_1 or 7) were present in all zones and seasons. Among the top 10 ASV-rich genera, *Cenococcum*, *Cortinarius*, *Russula*, and *Sebacina* were present in all zones, seasons, and sample types (Fig. 3c, Fig. S5c, Fig. S6). However, some genera preferred a certain elevational zone, season, or sample type. For example, *Cortinarius* and *Sebacina* were the ASV-richest genus (the genus that contains the largest number of ASVs among all genera) at high (41H) and low altitudes (29H, 31H, 33H, 35H), respectively. *Cortinarius* and *Russula* were the ASV-richest genera in the dry season, whereas *Cenococcum* was the ASV-richest genus in the wet season (Fig. S6). *Lactarius* was the ASV-richest genus in root samples, whereas *Inocybe* was the ASV-richest genus in soil samples (Fig. S6).

For both root and soil samples, the sampling zone explained more variations in richness (Table 1; root: zone 34.8%, season 20.5%; soil: zone 65.8%, season 3.7%) and composition (Table S3; root: zone 19%, season 3%; Soil: zone 43%, season 7%) than season.

**TABLE 1:** The effect of seasons and elevation zones on EcM fungal richness detected by two-way analysis of variance^*a*^

	Root samples			Root samples by plot			Soil samples		
Variables	Richness	Chao1	Shannon	Richness	Chao1	Shannon	Richness	Chao1	Shannon
Zone	0.348*	0.234*	0.192*	0.669*	0.636*	0.133	0.658*	0.614*	0.693*
Season	0.205*	0.155*	0.285*	0.193	0.157	0.322*	0.037	0.009	0.026

a The values are R^2^ representing the variation in richness that can be explained by season or altitude. Asterisks indicate the significance at the level of *p *< 0.05.

**FIG 4:**
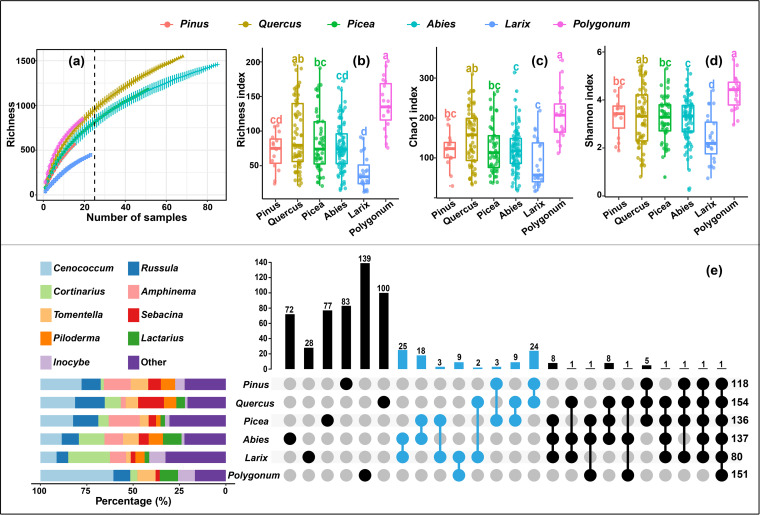
Diversity and shared or exclusive ASVs of EcM fungi in six host genera: *Pinus*, *Quercus*, *Picea*, *Abies*, *Larix*, and *Polygonum*. (a) Species accumulation curves based on root samples of each host genus. (b to d) The richness, Chao1, and Shannon indexes of the six host genera, respectively. The different lowercase letters represent significant differences (by Tukey HSD) at a level of *P* < 0.05. (e) Tableau stacked bar charts and upSetView plots based on ASVs (relative abundance > 0.1%) to display the relative number of ASVs in each genus and numbers of shared and exclusive ASVs among host genera. Host genera with overlapping distribution zones are highlighted in sky blue.

Furthermore, the EcM fungal richness also significantly varied among different host genera; *Quercus* and *Polygonum* hosted the richest EcM fungal diversity followed by *Picea*, *Abies*, *Pinus*, and *Larix* (Fig. 4a to d). Additionally, a total of 555 EcM fungal ASVs with >0.1% relative abundance were generated, and approximatively 91% of them were exclusively found in a single host genus (Fig. 4e). *Cenococcum*, *Inocybe*, and *Tomentella* in *Polygonum*; *Russula*, and *Sebacina* in *Quercus*; and *Cortinarius* in *Larix*, were the ASV-richest genera (Fig. 4e).

### Explanatory factors for spatiotemporal variations in EcM fungal community.

**FIG 5:**
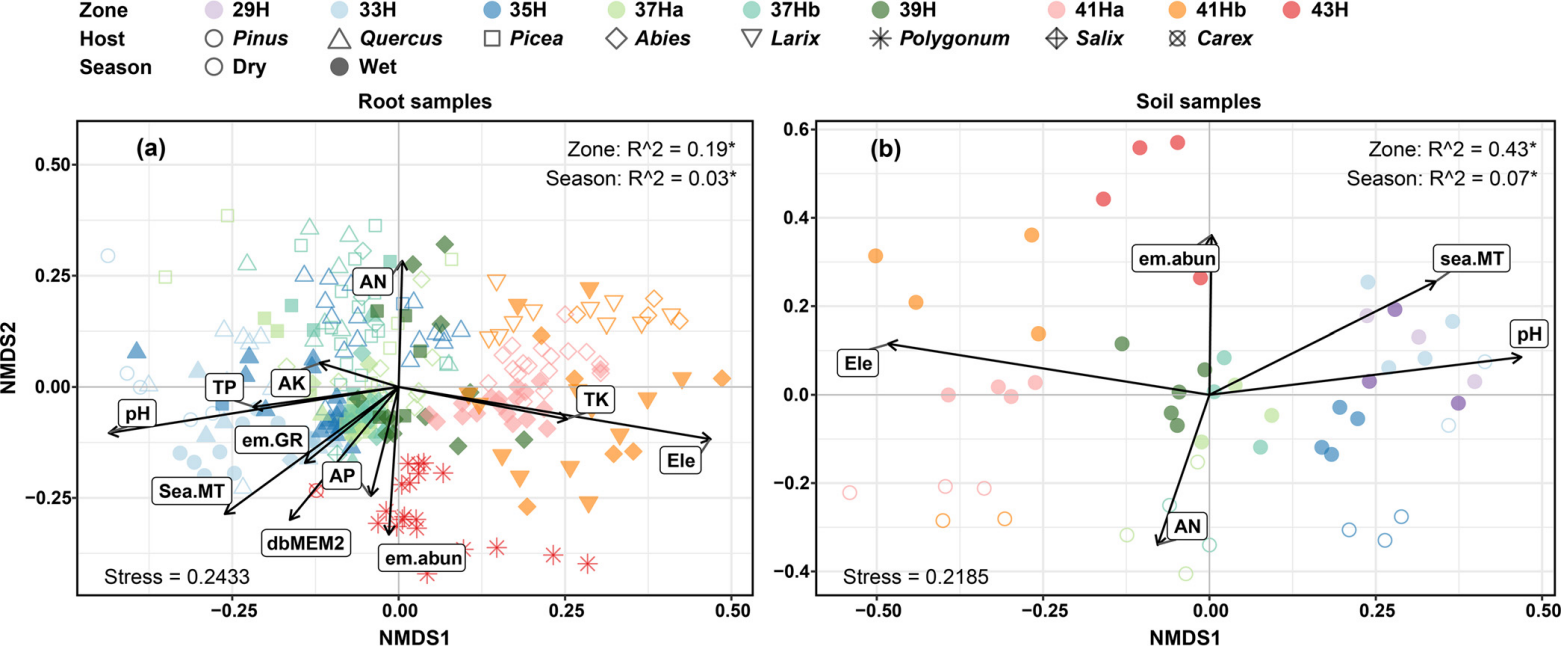
Nonmetric multidimensional scaling ordinations of the Bray–Curtis dissimilarity matrices of EcM fungal communities from root (a) and soil (b) samples. Arrows show the strength of correlation and direction of maximal increase for environmental variables with a significant contribution (*P* < 0.05) to community dissimilarities. Numbers after “Zone” and “Season” are R^2^, representing the variation in EcM fungal community composition that can be explained by altitude or season, detected using PERMANOVA. *, the significant level at *P < *0.05.

**FIG 6:**
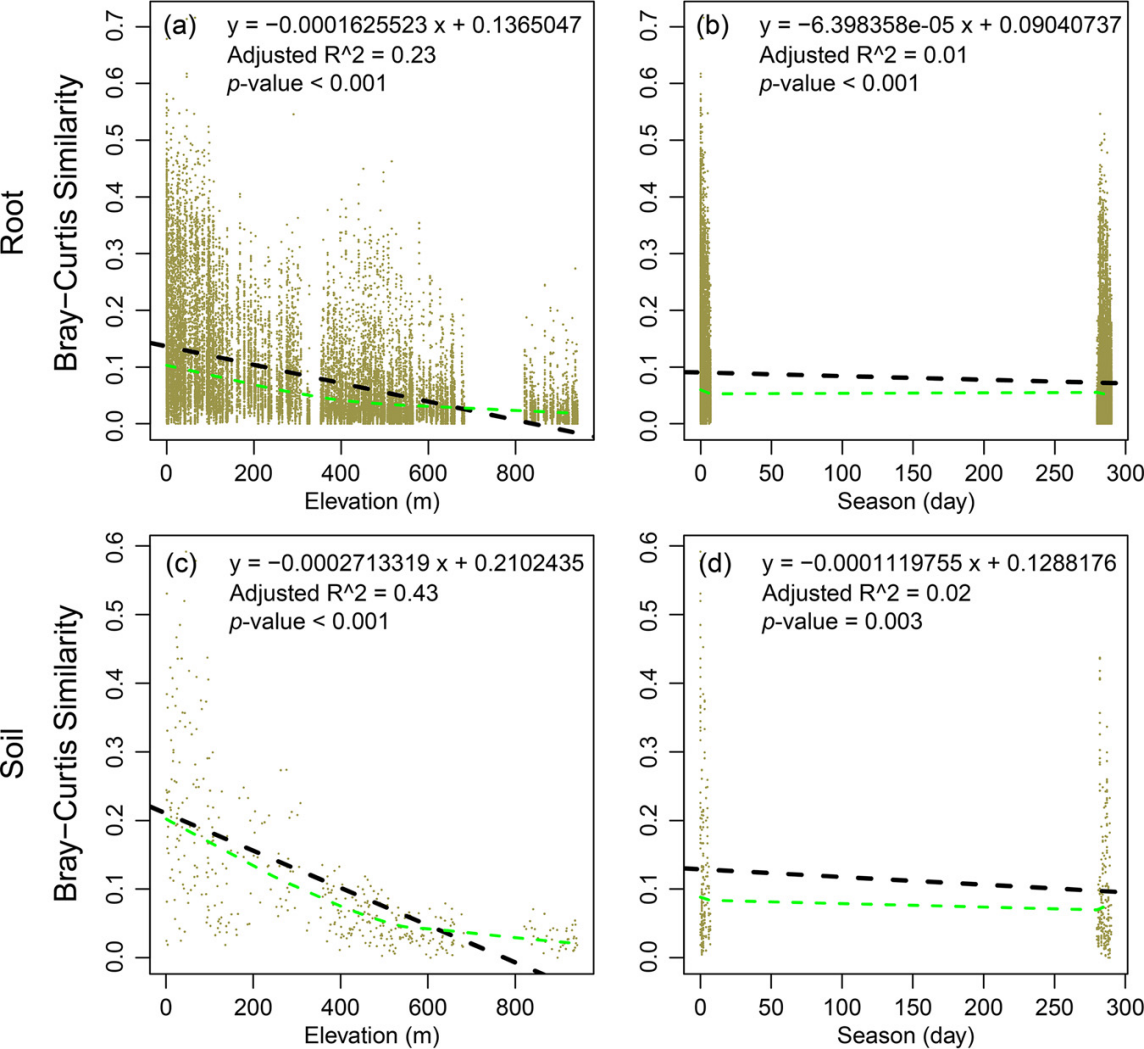
Linear regression model of community similarity with altitude interval matrix, and time interval matrix to conceptualize the relative importance of elevation and season. For the root samples (upper row), we make the two equations in graphs a and b equal, and put the 280-day time interval into the equation in graph b to calculate how much altitude interval would be required to capture the seasonal turnover. For the soil samples (lower row), the same method is used. The blue dash lines were obtained using the non-parametric smoother LOESS.

The results of stepwise multiple linear regressions showed that the distance-based Moran’s eigenvector maps (dbMEM) of spatial variables explained most variations in EcM fungal richness along different elevations (Table 2). The nonmetric multidimensional scaling (NMDS) and permutation-based multivariate analysis of variance (PERMANOVA) analyses showed significant elevation, season, and host separation of the EcM fungal community composition (Fig. 5, Table S3). The first axis of NMDS identified elevation as well as pH, total phosphorus (TP), or total potassium (TK) as the major determinants of the EcM fungal community composition. The second axis mainly correlated with sea.MT, alkaline-hydrolysable nitrogen (AN), or AP (Fig. 5). Further, we detected that changes in community similarity between dry and wet seasons (i.e., 280 days) were equivalent to the community turnover with an altitude interval of about 400 m (i.e., 394 m for root samples, 416 m for soil samples; Fig. 6).

**TABLE 2:** Stepwise, multiple linear regressions based on root samples and soil samples in dry and wet seasons to explore multivariate explanations for observed elevational patterns of EcM fungal richness

Sample types	Season	Variable^*a*^	Slope	SD	*t*	*p*	Independent contribution (%)	Adj.R^2^	*p*
Root	Dry	dbMEM1	89.51	12.6	7.11	<0.01	66.22	0.77	<0.01
		em.GR	−190.46	49.45	–3.85	0.02	10.61		
	Wet	dbMEM2	−57	11.34	–5.03	<0.01	50.95	0.62	<0.01
Soil	Dry	dbMEM1	44.44	5.65	7.87	<0.01	63.36	0.89	0.01
	Wet	pH	245.13	31.91	7.68	<0.01	49.17	0.71	<0.01
		dbMEM1	−16.85	5.01	–3.36	0.01	17.23		

a dbMEM, spatial eigenvectors generated from geographic coordinates (latitude and longitude); em.GR, richness of EcM plants at generic levels; pH, soil acidity and alkalinity.

**FIG 7:**
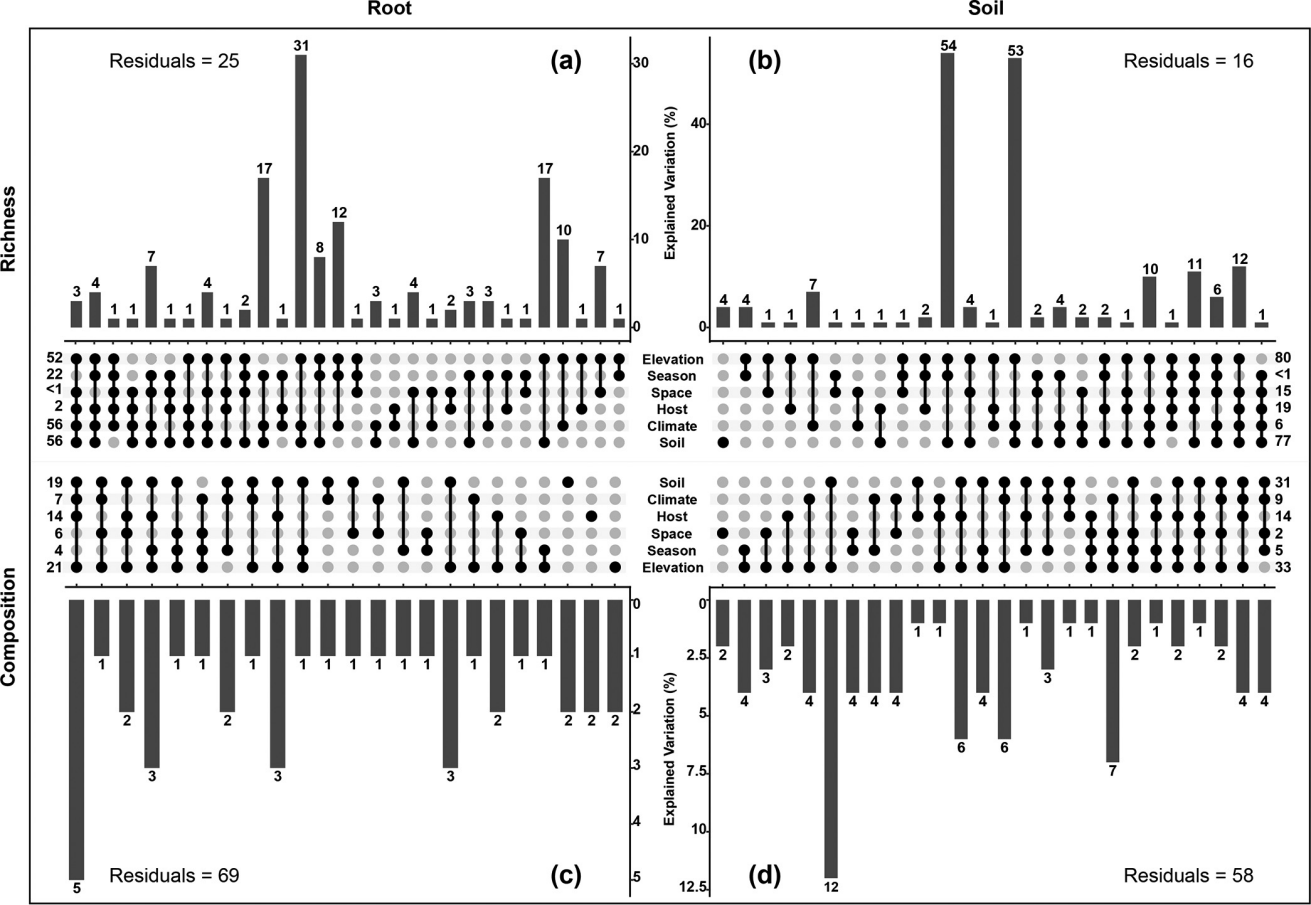
UpSetView plots of variation-partitioning results to show the pure and shared contributions of elevation, season, spatial (dbMEM1 and dbMEM2), host (em.GR, em.abun), climate (sea.MT), and soil variables (pH, TP, TK, AN, AP, and AK) on EcM fungal richness variations (a and b) and community composition variations (c and d) in root and soil samples, respectively. The numbers in the graphs are the percentage of variance explained by the corresponding environmental factors. The dot matrix and the corresponding bar above it show the values of shared and exclusive contributions. Negative values due to adjustment of R-squared mean negligible contributions and are not shown in the graph, but they are included in the computation of the total contribution of each variable category which were shown on the edge of the dot matrix. Residuals represent the percentage unexplained by these variables.

Variation partitioning showed that elevation explained the largest variances of EcM fungal community (i.e., 52%, 80%, 21%, and 33% in root-richness, soil-richness, root-composition, and soil-composition, respectively), followed by soil factors (56%, 77%, 19%, and 31% in root-richness, soil-richness, root-composition, and soil-composition, respectively) (Fig. 7). Meanwhile, the slow-changing environmental variables were not the main drivers of EcM fungal altitude variation in that they only explained a little proportion of it (< 0.1%, < 0.1%, 4%, and 12% in root-richness, soil-richness, root-composition, and soil-composition, respectively) (Fig. S7a, c, e, g), while the fast-changing environmental variables were the main drivers of EcM fungal seasonal variations as they explained considerable proportion of the variations observed in season (82%, 7%, 33%, and 45% in root-richness, soil-richness, root-composition, and soil-composition, respectively) (Fig. S7b, d, f, h).

## DISCUSSION

### High EcM fungal diversity on Baima Snow Mountain.

This study identified 3,416 ASVs (691 OTUs) and estimated 3,932 ASVs (725 OTUs) of putative EcM fungi. To the best of our knowledge, this is one of the highest richness reported from a single mountain area (14, 15, 17, 20, 51). The high EcM fungal richness can be attributed to: (i) the use of high-throughput sequencing (HTS) on amplicons; and (ii) the collection of soil and root samples from forest and alpine meadow zones in both wet and dry seasons in a biodiversity hot spot. Gao and Yang (49) detected 23 EcM fungal OTUs from the root tips of *Polygonum macrophyllum* from Baima Snow Mountain using Sanger sequencing (summarized from Table II in Gao and Yang [49]). In the present study, we identified 182 EcM fungal OTUs from *Polygonum* roots at a single elevation level (approximately 4,450 m) on Baima Snow Mountain using HTS. This considerably improves our understanding of the richness of EcM fungi associated with *Polygonum* species in the region. Concerning the forest zone, using Sanger sequencing, 302 and 200 EcM fungal OTUs were detected from Mt. Fuji and Mt. Ishizuchi, respectively (17, 31). These are less than the EcM fungal OTUs detected in the present study (466 and 373 OTUs collected from root samples in wet and dry seasons, respectively). Notably, steep species accumulation curves of soil (Fig. S3a) indicated insufficient sampling of soil that may have resulted in the lower number of ASVs detected in soil than that in root (Fig. S3e). In contrast, previous studies have reported a higher number of EcM fungal richness in soil than that in root in Guadarrama (Spain), Pyrenees (Spain), Vosges (France), European Alps, and Changbai Mountain (China) (28, 33, 52). Thus, the EcM fungal richness could have been underestimated in the present study.

### Spatiotemporal dynamics of the EcM fungal community on Baima Snow Mountain.

In the forest zone, a monotonically decreasing richness pattern were observed (Fig. 2). This pattern has been reported in many other distant temperate regions, such as Hyrcanian forest of northern Iran (12), Nahuel Huapi National Park of Argentina (53), and Changbai Mountain of northeast China (52). Harsher climate conditions such as lower temperature, stronger wind, and UV radiation may decrease EcM fungal diversity at higher elevations (12, 52). Further, the extinction of species that is less competitive in harsh climatic conditions could also be a reason for the declining pattern (12), which could be also inferred from our results—a declining trend was observed in the number of exclusive EcM fungi in each zone of the forest with increasing elevation (Fig. 3a and b, Fig. S5a and b).

In the alpine meadow zone, a significantly higher EcM fungal richness was observed than in the adjacent forest zone. Such cross-zone variations have been reported in Canadian Rockies—however, it showed a reverse trend, where a decrease in EcM fungal richness in the alpine zone was detected relative to that of the adjacent subalpine forest zone (54). Meanwhile, the EcM fungal community compositions in the alpine meadow zone also showed significant differences compared with that in the adjacent forest zone (Fig. 5, Table S3). It is not surprising that *Polygonum* plants in the alpine meadows of Baima Snow Mountain harbored an abundant EcM fungal richness. Studies in different alpine or arctic habitats have reported 300–600 EcM fungal OTUs from *Bistorta vivipara* (33, 36, 54). Meanwhile, the cross-zone variation in EcM fungal richness and compositions can be ascribed to the cross-zone variations in microenvironment (54), such as temperature; richness and abundance of EcM plants; and soil pH, TP, and AN; these variations were observed in the present study (Fig. 1).

Soil and root samples have been widely used in EcM community studies, but only a few studies have simultaneously evaluated both the sample types (28, 33, 52). In the present study, although the EcM fungal richness in the root and soil samples showed similar pattern with increasing elevation (decline in the forest zone, and an increase at the alpine meadow zone), the seasonal patterns observed in soil and root samples were different. The highest EcM fungal richness detected from root samples in the wet season can be attributed to the high carbon productivity and host allocation in this season (30, 55), whereas a more abundant and diverse spore bank in soil after the fruiting season may be responsible for the highest EcM fungal richness detected in soil samples in the dry season (36). This heterogeneity suggests that different underlying factors may affect EcM fungal richness in different sample types. Therefore, sampling both root and soil simultaneously is helpful to better understand the spatiotemporal patterns of EcM fungal richness. In contrast, the EcM fungal community compositions showed similar strong spatiotemporal turnovers regardless of the sample type (Fig. 3, Fig. 5, Fig. S5), which has been reported by several other studies for different altitudes (12, 15, 17, 54) and seasons (34, 37).

### Soil property as one of the key factors driving spatiotemporal variations in EcM fungal community.

Despite significant elevation and seasonal variations in EcM fungal richness (Fig. 2, Table 1) and strong turnovers of community compositions (Fig. 3, Fig. 5, Fig. S5, Table S3), elevation and season showed no or nearly negligible pure effects on such dynamics. Besides, soil, host, climate, and space also showed the same trend (Fig. 7), which indicated that these factors could interact in complex ways to jointly influence the EcM fungal community (20, 31, 32). Increasing elevation can decrease the temperature and change the composition of host (12), while dry season at Baima Snow Mountain has low temperature, soil moisture, and photosynthate, and high litter input. These factors can affect EcM fungal community by changing the soil productivity, microbial heterotrophic activities, ecological interactions, and the quantity and quality of resources (12, 24, 39).

Despite this, consistent with several local and regional studies (56–59), soil property showed high total effects in shaping EcM fungal assemblages (Fig. 7). Particularly, soil pH may play a central role in the assembly of the EcM fungal community along an altitude gradient (13, 16) because it is an excellent integrator of soil nutrient availability (59). For instance, it can affect the exchange capacity of anions and cations, and a decrease in pH can reduce the biological availability of N and P (60, 61). Additionally, the availability of N and P can impose habitat filtering on EcM fungal communities (6, 7, 62). For example, under a long-term N deposition, the community structure can shift from taxa that are beneficial for N uptake in a low-N environment to taxa with a good ability to take up P in a N-rich environment (6, 7).

When considering only the richness pattern along the altitude gradient, spatial dbMEM vectors and not edaphic factors had the greatest influence on EcM fungal richness along the elevation gradient (Table 2). Two non-exclusive causes could explain this observation. First, all detected soil factors together rather than a single soil factor determine the altitude variation in EcM fungal richness. This results in a few soil factors being retained in the final model of multiple linear regression. In fact, these soil factors all varied significantly along the altitude with considerable R^2^ values (Fig. 1); thus, these variables may equally contribute to richness variation along an altitude gradient, making it difficult to distinguish the factors having the greatest effect. Second, dbMEM has an important role in affecting the EcM fungal richness along an altitude gradient compared with a single soil factor. This suggests that spatial processes, such as dispersal limitation, may determine the EcM fungal richness pattern along the altitude on Baima Snow Mountain.

Furthermore, significant separation of EcM fungal communities were observed among different host genera (Fig. 5a, Table S3), which indicated the importance of host in determining the EcM fungal community (17, 63–65). The host specificity (63) could be a cause of this separation. Because each host genus possessed multiple exclusive ASVs, and some EcM fungal groups also showed host preference, such as ASVs of *Cenococcum* and *Cortinarius* were more often detected in *Polygonum* and *Larix*, respectively (Fig. 4e). Given the importance of host and the co-variation of host community and altitude, considerable effects of the hosts on the altitudinal variations in EcM fungal community should be expected. In fact, host only contributed minor effects (1% to 2%) to variations with elevation, whereas soil contributed more effects to the variations with elevation, followed by climate (Fig. 7). This further illustrates the high importance of soil in affecting the elevational variation of EcM fungal community.

### Variations in environmental factors drive greater spatial variability than seasonal variability in EcM fungal community.

Similar to the results of local- and regional-scale studies on soil bacteria and fungi (29, 41, 66), our study revealed that spatial variability exceeded seasonal variability on EcM fungal richness (Table 1) and community compositions (Fig. 5, Table S3) in Baima Snow Mountain. Although seasonal variability might be masked by the relic DNA (DNA released from dead microbes) in soil (67), similar results based on live root samples convinced us that spatial variations in the EcM fungal communities were higher than seasonal variations. In addition, the situation could be that the actual seasonal variability of EcM fungal community was lesser than the detected variability in the study area. This could be a result of the sampling strategy as different plots and trees were sampled in the dry and wet season, thereby introducing a locality effect, which might inflate the seasonal variability. However, relative to the season, the locality effect in this study would be negligible because the seasonal turnover of soil fungal communities would be equivalent to the community turnover observed over thousands of kilometers (68). In each elevational zone, the horizontal distance between the sampling plots of wet season and plots of dry season was only 100 m at most in the present study. These observations indicate that elevation is more important than contrasting seasons in shaping the heterogeneity of EcM fungal community, which is consist with our first hypothesis.

It is not surprising to observe such spatiotemporal variability as the present study spanned an altitude gradient (c. 1,500 m), which was much larger than the altitude interval (c. 400 m) that could capture the EcM fungal community turnover between the dry and wet seasons (Fig. 6). In addition, all edaphic factors (except for AK), which were shown to be the most important factors shaping the spatiotemporal dynamics of EcM fungal richness and community composition (Fig. 7, see also discussion above), showed significant variations along the elevation gradient. In contrast, only AP showed a marginally significant change between seasons (Fig. 1). Further, host and climatic factors also significantly changed along the altitude rather than between seasons, and soil, host, and climate explained more elevational variance of EcM fungal community than seasonal variance (Fig. 7). All these results indicate that more variations in environmental factors along the altitude gradient contribute to a greater variability in the EcM fungal community across different elevations than across seasons.

Elevation characteristics are usually characterized by specific temperature and slow-changing soil properties, such as soil pH and total soil nutrients—organic carbon, N, P, and K are stable with respect to time and take centuries or millions of years to change (41). Temperature is a key driver that shapes the EcM fungal assemblages at the regional scale (4, 69), whereas slow-changing soil properties show an explanatory power on spatial dynamics of soil bacterial and fungal communities (41). However, only a small portion of the variations in the EcM fungal community along the altitudinal gradient could be explained by these variables (Fig. S7a, c, e, g). Instead, other factors, such as em.GR, em.abun, AN, AP, and AK, jointly affected the altitudinal variations. This implies that several environmental factors work together to shape the EcM fungal community along the altitudinal gradient. Interestingly, the fast-changing environmental variables, namely, sea.MT, AN, AP, and AK, could explain considerable variations in the EcM fungal community between seasons (Fig. S7b, d, f, h). Seasonal temperature changes can influence the EcM fungal community by affecting the litter degradation and carbon allocation of host plants (30), and the AN, AP, and AK are fast changing soil properties that can drive the seasonal dynamics of soil bacterial and fungal communities (41). This further shows that the seasonal niche differentiation is smaller than the elevational niche differentiation, which leads to less seasonal variations in EcM fungal communities compared to altitudinal variations.

In conclusion, we explored the spatiotemporal patterns of the EcM fungal community on Baima Snow Mountain by simultaneously studying the soil as well as root samples. An abundant richness of EcM fungi was detected in this region. Except the difference of the seasonal patterns of EcM fungal richness observed in soil and root samples, the EcM fungal richness showed similar pattern with increasing elevation (decline in the forest zone, and an increase at the alpine meadow zone), and the EcM fungal community composition showed strong turnovers across elevational zones and between seasons, regardless of the sample types. Elevation is more important than season in shaping the heterogeneity of EcM fungal community. This may be attributed to the larger niche differentiation along large altitude gradient than between season. The influence of altitude and season on the EcM fungal community could not be explained by considering altitude or season separately, but it could be explained by analyzing the combined effects of several environmental factors, particularly soil variables. This indicates that environmental filtering, resulting from elevational and seasonal niche differentiations, is the underlying mechanism for spatiotemporal dynamics of the EcM fungal community. Notably, spatiotemporal dynamics in the EcM fungal community and their underlying factors may differ at different geographical scales. Therefore, the local scale findings of the present study should be further tested at regional and global scales.

## MATERIALS AND METHODS

### Study area.

Baima Snow Mountain (28°35′–27°24′N, 98°55′–99°24′E) is located between Deqin and Weixi counties in Yunnan Province of southwestern China. It has a cold temperate, montane monsoon climate characterized by distinct dry (November to April) and wet (May to October) seasons. Using the 3-year (2016 to 2018) meteorological data from six weather stations and temperature lapse rate, the annual mean temperature (AMT) and mean temperature during dry (DMT) and wet (WMT) seasons were estimated to be 11.8°C to −1.5°C, 7.3°C to −6.2°C, and 16.3°C to 3.2°C, respectively, at an altitude of 2,900 m to 4,500 m above the sea level. The mean precipitation range over 7 months (from April to October) was 385 mm to 666 mm from 2,900 m to 4,500 m. The soil type is brown montane soil that develops on granite (70, 71), which is rich in OM with a pH < 7 (72).

### Sampling design.

Sampling was conducted along the elevation gradient (2, 900 m to 4,500 m) in the eastern slope of Baima Snow Mountain during both dry (November 2017) and wet (August 2018) seasons after studying community compositions of the host plants of EcM fungi. The sampling area covered both forests, one region from 2,900 m to 4,200 m with a dominance of different EcM plants, such as *Pinus*, *Quercus*, *Picea*, *Abies*, and *Larix*, and the alpine meadow with *Polygonum macrophyllum* as the dominant EcM plant (above 4,300 m). Briefly, we set 10 sampling zones at eight different elevation levels, namely, 29H, 31H, 33H, 35H, 37H, 39H, 41H, and 43H, which roughly indicate 2,900 m, 3,100 m and so on. In each zone, two to five plots (20 m × 20 m) were set. Two sampling zones (marked as a and b) were set at 37H and 41H, respectively, to assess the heterogeneity of EcM plant compositions at two elevation levels. It should be noted that the same plots and trees were not sampled in the dry and wet season because of the coordinate positioning deviation. However, the geographic coordinates of the dry season plots were noted to ensure that the same plots and trees were sampled in the two seasons. Information on the sampling zones is detailed in Table S1. The study area, distribution of sampling plots, and vertical vegetation zones are shown in Fig. S1. For each zone from 3,300 m to 4,300 m, the root samples of EcM plants were collected from two to three plots, and soil samples were collected from all plots. For the other two zones, namely, 29H and 31H, only soil samples were collected as the soil was too dry for the collection of root samples. Due to harsh environmental conditions in the dry season, we conducted sampling only at 33H, 35H, 37Ha, 37Hb, 41Ha, and 41Hb in this season. In total, sampling in the dry season covered 15 plots in six zones, whereas that in the wet season covered 37 plots in 10 zones (Table S1).

In each plot, nine soil cores (4.5 cm in diameter and 10 cm in depth) were collected from each of the two diagonals. These soil cores were mixed thoroughly, sifted using a 2-mm sieve, and then air dried to obtain the soil sample. To collect root samples, five to eight EcM trees that had similar diameters at breast height were selected based on the relative abundance of different EcM tree genera. During this procedure, the selected trees were evenly distributed in the plot. For each tree, a root sample was collected by tracing the roots from the base of the trunk in two directions. The root samples were transferred to the laboratory, where they were stored at 4°C. Each root sample was gently washed with water to remove the soil attached to the surface. Thereafter, no less than 200 EcM root tips were picked under a stereomicroscope, and they were dried with silica gels to obtain an EcM sample. For *Polygonum*, five soil cakes (approximately 20 cm × 20 cm × 10 cm) with *Polygonum* plants were collected from two equidistant diagonals. We picked root tips from five individual plants in the same soil cake to prepare an EcM sample. For ease of understanding, the root samples mentioned in the manuscript refer to EcM samples unless otherwise specified.

### Collection of environmental variables.

AMT, DMT, and WMT for 3 years (January 2016 to December 2018) were obtained from six weather stations set along the elevation gradient of Baima Snow Mountain. The soil properties were measured in Yunnan Sanbiao Agriculture and Forestry Technology Co., Ltd. (Kunming, Yunnan, China) according to the standards of the agriculture or forestry industry of China. Soil pH was determined in a 2.5:1 (m/w) water/soil suspension using a pH meter (NY/T 1377–2007). Soil OM content was determined by the adjusted potassium dichromate method (NY/T 1121.6–2006). Soil TN content was determined using the semi-micro Kjeldahl method (NY/T 53–1987). TP content was determined by molybdenum-blue colorimetry (NY/T 88–1988). TK content was determined by flame photometry (NY/T 87–1988). The AN content was determined using the alkaline-hydrolysable diffusion method (LY/T 1229–1999). The AP was determined using the molybdenum-blue method (NY/T 1121.7–2014). The AK was extracted by ammonium acetate and then determined by flame photometry (NY/T 889–2004). The abundance (em.abun) and richness of EcM plants at genus and family levels (em.GR and em.FR) were counted for each plot. The distance-based Moran’s eigenvector maps (dbMEM) were constructed from spatial coordinates (latitude and longitude) using the “dbmem” function of adespatial package (73) in R v.3.6.1 (74). The geographical coordinates and values of all variables are detailed in Table S1.

### Molecular work.

The DNAs of 52 soil samples (15 and 37 samples from dry and wet seasons, respectively) and 263 EcM samples (109 and 154 samples from dry and wet seasons, respectively), as well as two negative controls (roots of *Quercus* sp. and *Abies* sp.) and two positive controls (fruitbodies of *Leccinum* sp. and *Russula* sp.) were extracted using an E.Z.N.A Soil DNA kit (Omega Biotek, Norcross, GA, USA). The internal transcribed spacer 2 (ITS2) region was amplified for each sample using a two-step PCR protocol (modified from Matsuoka et al. [20]). First, the entire ITS region was amplified using the fungus-specific primer ITS1F (75) and universal primer ITS4 (76), and the PCR products were diluted to 20 ng/μL (no dilution was performed when the concentration was less than 20 ng/μL). Second, the ITS2 region was amplified with the universal primer pair ITS3 (76) and ITS4 using the diluted PCR products as templates in the first step. The library preparation and paired end (300 bp) Illumina MiSeq sequencing was conducted by Personal Biotechnology Co., Ltd. (Shanghai, China). Raw sequences are deposited in the Sequence Read Archive under Bioproject PRJNA720443.

### Bioinformatic analysis.

Cutadapt v2.10 (77) was used to trim barcodes, primers, and low-quality ends from demultiplexed reads (parameters: -q 20 -e 0 –no-indels –discard-untrimmed –pair-filter any). Subsequently, the paired-end reads were merged and then assigned to unique sequences using vsearch v2.14.1 (78). The unique sequences were denoised using the unoise algorithm (79) in usearch v10.0.240 (80) to remove chimeras and to obtain ASVs (81). For comparison with other OTU-based studies, unique sequences with a 97% identity were clustered as OTUs using the “cluster_otus” command in usearch. The ITS2-only region was extracted from ASV sequences using ITSx 1.1.2 (82), and it was blasted against the UNITE database (83) using vsearch v2.14.1 to identify their taxonomic ranks. Non-fungal ASVs were removed using a script shared by Zhang et al. (84). Subsequently, we normalized each sample to 10 000 reads.

FUNGuild v1.1 (85) was used to extract the EcM fungi from ASVs. The ASVs parsed as EcM using FUNGuild were extracted as EcM fungi candidates. Among them, those with a “confidence ranking” of “highly probable” were directly assigned as EcM fungi while those with a “confidence ranking” of “possible” or “probable” were blasted against the UNITE database, and the ones having the best match to an EcM taxon with > 97% identity were assigned to EcM fungi. The ASVs were also searched against a list obtained from Tedersoo and Smith (86), which includes EcM fungal groups identified later than the publication of FUNGuild, to extract EcM fungal ASVs not parsed via FUNGuild. Subsequent analyses of alpha and beta diversities were based on the EcM fungal ASVs.

### Statistical analyses.

All statistical analyses were performed using R v.3.6.1 (74). The Bonferroni method was used for multiple testing correction to obtain the adjusted *P* value. All graphs, except for the upSetView plots, were plotted in R using the built-in functions or the ggplot2 package (87). Normal distribution of each variable and pairwise correlations between variables were assessed using “chart.Correlation” function in PerformanceAnalytics package (88). Data with nonnormal distribution were square-root or log transformed. Four variables, namely, em.FR, AMT, OM, and TN, were removed because of their high correlation (*r* > 0.7) with others (Fig. S2). The homogeneity of variances of variables were assessed using Bartlett’s test (89). The relative importance of elevational zones and seasons were evaluated using two-way analysis of variance (ANOVA) for explaining the variations in variables. If the variance of a variable is homogeneous, the “aov” function in the stats package was used; if the variance was not homogenous, the “scheirerRayHare” function in the rcompanion package (90) was used. The altitudinal and seasonal changes of variables are shown in Fig. 1.

The species accumulation curves and Chao estimators (91) were evaluated using “specaccum” and “specpool” functions in the vegan package (92) to estimate the EcM fungal population size of Baima Snow Mountain. To explore the EcM fungal *α*-diversity patterns along the altitude gradient between seasons, the observed richness, Chao1 index (91), and Shannon index (93) were calculated for root and soil samples. Two separate analyses were conducted for root samples—one based on single root samples and another based on the root samples merged via a plot. The difference in diversity among different elevation zones and seasons was detected using “aov” and “TukeyHSD” functions. As the richness observed in forests seems to show a unimodal pattern along the altitude gradient, both quadratic and linear regression analyses were used to examine relationships between richness and elevation. The best regression model was guided by the lowest AICc (94). The relative importance of elevational zones and seasons to explain the variations in EcM fungal richness was evaluated using ANOVA analysis. ASVs with a relative sequence abundance > 0.1% were selected to draw stacked graphs at the genus level and upSetView plot graphs or Venn diagrams using TBtools v1.046 (95) to observe the exclusive and shared ASVs.

Stepwise multiple linear regression was used to explore multivariate explanations in the observed elevation patterns of EcM fungal ASV richness. If the best model of multiple regression contained more than one variable, the “hier.part” function in Hier.part package (96) was used to explore the independent contribution of each variable. Hellinger-transformed community matrix was used to calculate the Bray–Curtis dissimilarity distances (97) using “decostand” and “vegdist” functions in the Vegan package. Community dissimilarities were ordinated NMDS using the “metaMDS” function. The contribution of elevation zones and seasons to the EcM fungal community structure was assessed via PERMANOVA using the “adonis” function. To better conceptualize the relative importance of elevational and seasonal predictors, we fitted a linear regression model of community similarity (1 minus Bray–Curtis dissimilarity distances) with altitude interval matrix, and time interval matrix for sample collection, between all pairs of samples, respectively. Based on the models, we calculated how much altitude interval would be required to capture the seasonal turnover observed 280 days apart. The altitude interval matrix and time interval matrix were obtained from the altitude and collection time of each sample using “vegdist” functions.

To explore the relative importance of individual environmental variables on the composition of EcM fungal community, the “envfit” function was used to fit environmental variables to the NMDS results based on 999 permutations. To quantify the relative importance of elevation, season, space (dbMEM1 and dbMEM2), host (em.GR and em.abun), climate (sea.MT, i.e., DMT and WMT), and soil (pH, TP, TK, AN, AP, and AK), on shaping the structure of EcM fungal richness and composition, variation partitioning analysis was performed using the rdacca.hp package (98). Following this, variations in EcM fungal community were then partitioned among elevations, slow-changing environmental factors (pH, TP, and TK), and other factors; and among seasons, fast-changing environmental factors (sea.MT, AN, AP, and AK), and other factors, using the “varpart” function in the vegan package to explore the underlying mechanism of spatiotemporal dynamics of EcM fungal community. As the host genus-level identity of each root sample can be determined, we can explore the influence of host genus-level identity on the EcM fungal community using similar methods mentioned above. Normalization was conducted using the host genus with the lowest number of samples as the normalizing size when we compared the community diversity among host genera.

The study has two limitations: (i) several zones had only two replicates, and hence, statistics analysis could not be performed for them, and (ii) there are fewer plots in dry season than those in wet season; the pairing of plots did not extend across all the elevational zones. This imbalance in the sample numbers could influence the results. To overcome these issues, the number of plots, soil samples and root samples in each elevation zone in the dry and wet seasons were all normalized to the same number during the ANOVA, PERMANOVA, and variation partitioning analyses.

### Data availability.

Raw sequences are deposited in the Sequence Read Archive under Bioproject PRJNA720443. The read count ASV table with the representative sequence and taxonomy of each ASV was provided as supplemental file 2, the corresponding metadata was provided as supplemental file 3.
