# Supplementary material for: Elevation Matters More than Season in Shaping the Heterogeneity of Soil and Root Associated Ectomycorrhizal Fungal Community
Source: Microbiol Spectr. 2022 Jan 12;10(1):e01950-21. doi: 10.1128/spectrum.01950-21 (PMC8754124; doi:10.1128/spectrum.01950-21)
Supplement: SUPPLEMENTAL FILE 1 — Supplemental material. Download SPECTRUM01950-21_Supp_1_seq13.pdf, PDF file, 1 MB [file spectrum01950-21_supp_1_seq13.pdf]

Microbiology Spectrum **Supplemental Material**

Elevation matters more than season in shaping the heterogeneity of soil and root associated ectomycorrhizal fungal community

Sai Gong,[a,b,c] Bang Feng,[a,b]# Si-Peng Jian,[a,b] Geng Shen Wang,[a,b,c] Zai-Wei Ge,[a,b] Zhu Liang Yang[a,b]#

The following supporting information is available for this study:

Fig. S1 The study area, distribution of sampling plots, and vertical vegetation zones on the eastern slope of Baima Snow Mountain.

Fig. S2 The pairwise correlations between environmental factors.

Fig. S3 Number of ectomycorrhizal (EcM) fungi ASVs detected in root and soil samples from the wet and dry seasons.

Fig. S4 Elevation and seasonal variations in EcM fungal Chao1 index and Shannon index.

Fig. S5 UpSetView plots (a-b) and tableau stacked bar charts (c) based on EcM fungal ASVs (relative abundance > 0.1%) from soil samples.

Fig. S6 Tableau stacked bar charts based on EcM fungal ASVs (relative abundance more than 0.1%) from root samples (a) and soil samples (b) in dry and wet seasons.

Fig. S7 Venn diagrams of variation-partitioning results to show pure and shared contributions of slow-changing and fast-changing environmental variables to elevation and season variations of EcM fungal communities.

Table S1 Geographic coordinates for sampling plots and their corresponding environmental variables.

Table S2 General descriptions of EcM fungi in different classification levels on Baima Snow Mountain.

Table S3 Significant effect of seasons, elevation zones and host genera identity on EcM fungal community composition detected by PERMANOVA.

Fig. S1 The study area, distribution of sampling plots, and vertical vegetation zones on the eastern slope of Baima Snow Mountain. Below 2600 m are the zones of warm and dry valleys colonized by shrubs and herbs. Between 2600 m and 4300 m are forests characterized by different dominant ectomycorrhizal (EcM) plants. Between 4300 m and 4800 m are alpine meadows with *Polygonum macrophyllum* as the main EcM plant. Above 4800 m are alpine screes. C-*Pin*, coniferous forest dominated by *Pinus* species (e.g. *P. densata*) is present at 2600–3000 m. C_B-*Pin*, coniferous forest (mostly *P. densata*) with scattered broad-leaved species (e.g. *Quercus semecarpifolia*) is present at 3000–3400 m. B_C-*Que*, broad-leaved forest (mostly *Q. semecarpifolia*) with scattered coniferous species (e.g. *Picea likiangensis*) is present at 3400–3600 m. C_B-*Pic*, coniferous forest (mostly *P. likiangensis*) with scattered broad-leaved species (e.g. *Q. semecarpifolia*) is present at 3600–3800 m. C-*Pic-Abi*, coniferous forest dominated by *Picea* species (e.g. *P. likiangensis*) or *Abies* species (e.g. *A. forrestii*) is present at 3800–4000 m. C-*Abi-Lar*, coniferous forest dominated by *Abies* species (e.g. *A. forrestii*) or *Larix* species (e.g. *L. potaninii*) is present at 4000–4300 m. ALM-*Pol*, alpine meadow with *Polygonum* species (e.g. *P. macrophyllum*) as the main EcM plant is present at 4300–4800 m. The altitude range marked in light gray is the sampling area of our study.

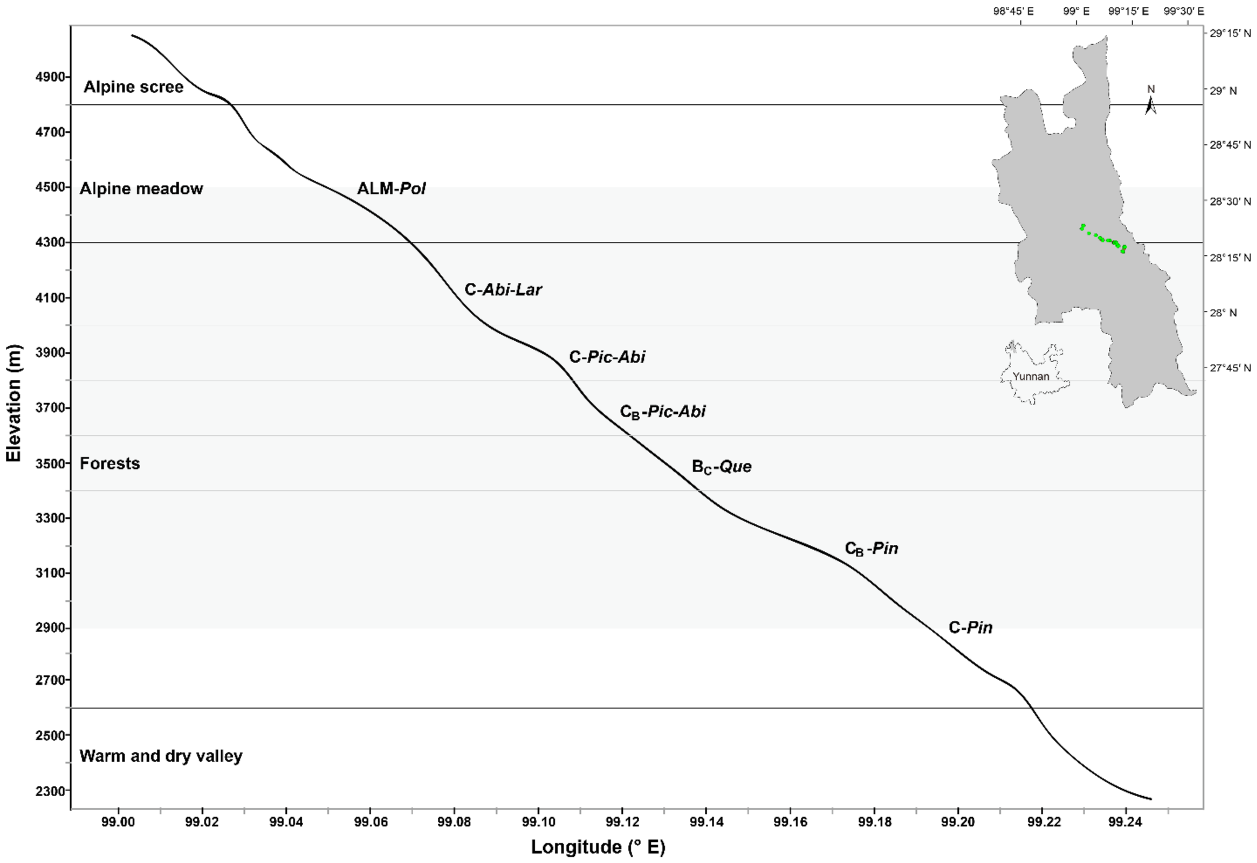

Fig. S2 The pairwise correlations between environmental factors. em.GR, richness of EcM plant at the genus level; em.FR, richness of EcM plant at the family level; em.abun, the number of individuals of each EcM genus; AMT, annual mean temperature; sea.MT, dry-season and wet-season mean temperature; OM, soil organic material; TN, total nitrogen; TP, total phosphorus; TK, total potassium; AN, alkaline-hydrolysable nitrogen; AP, available phosphorus; and AK, available potassium. Data of AMT and sea.MT were not transformed. Data of OM and TN were square-root transformed. Data of other variables were log transformed. Log transformation for em.GR, em.FR, em.abun, pH, TP, TK, AN, AP, and AK; square-root transformation for OM and TN. The numbers in the upper triangular matrix represent the correlation coefficient. One, two, and three asterisks indicate significant difference at the level of 0.05, 0.01, and 0.001.

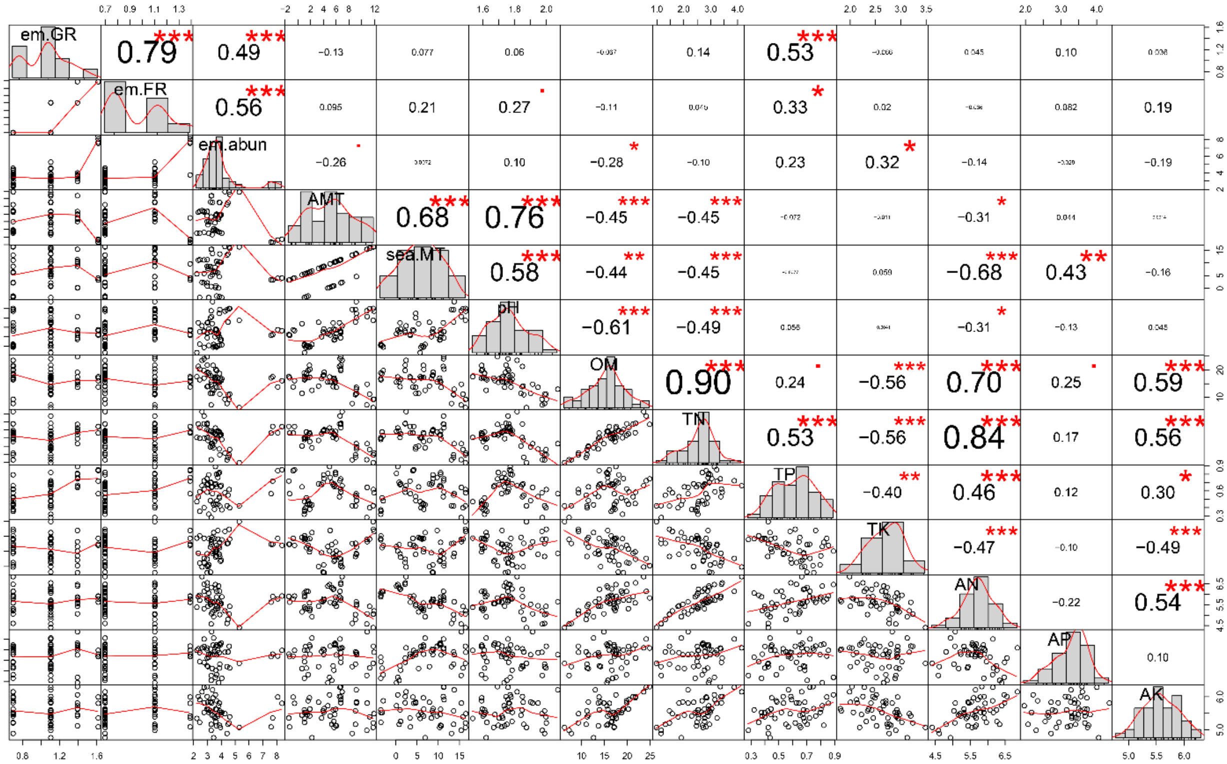

Fig. S3 Number of ectomycorrhizal (EcM) fungi ASVs detected in root and soil samples from the wet and dry seasons. a, species accumulation curves. b, observed richness (Observed) and estimated richness (Chao). c-d, Venn diagrams to show shared and exclusive ASVs between the dry and wet seasons from root and soil samples, respectively. e, Venn diagram to show shared and exclusive ASVs between root and soil samples. All, all root and soil samples; Allroot, all root samples of the two seasons; Allsoil, all soil samples of the two seasons; Root2017, root samples of dry season; Root2018, root samples of wet season; Soil2017, soil samples of dry season; Soil2018, soil samples of wet season.

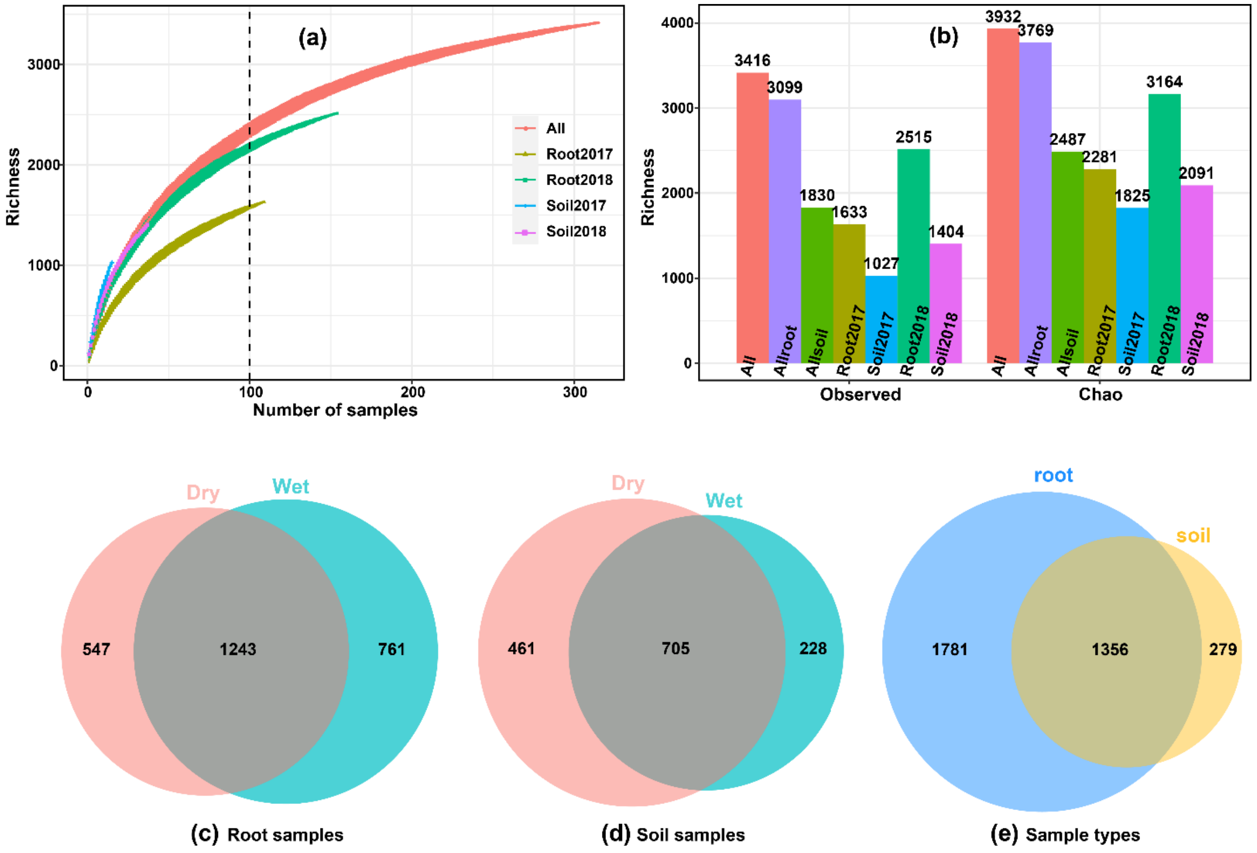

Fig. S4 Elevation and seasonal variations in EcM fungal Chao1 index and Shannon index. The left, middle, and right columns of graphs are based on single root samples, root samples merged via plots, and soil samples, respectively. Zones without letters on the top of boxes or short solid lines below boxes had the number of samples that did not meet the critira (n > 3) of statistical test; they were not included in the statistical analysis.

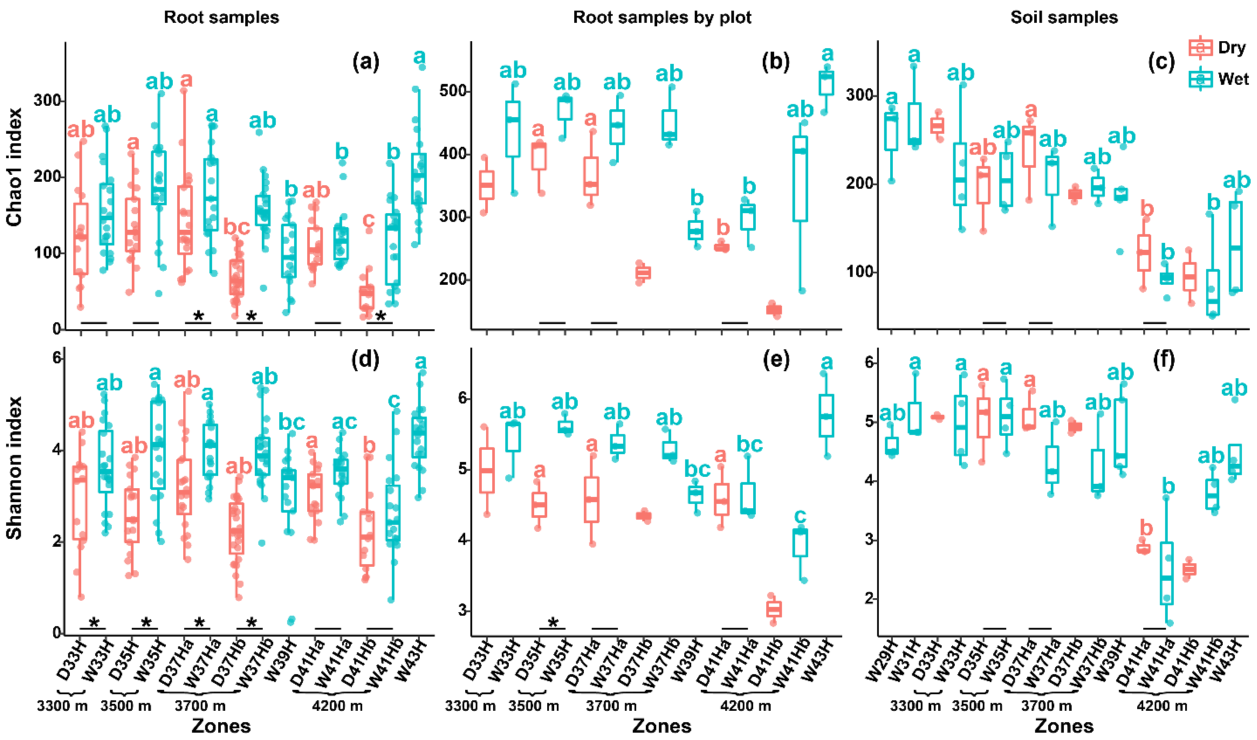

Fig. S5 UpSetView plots (a-b) and tableau stacked bar charts (c) based on EcM fungal ASVs (relative abundance > 0.1%) from soil samples to show the number of shared and exclusive ASVs among zones and the relative number of ASVs in each genus.

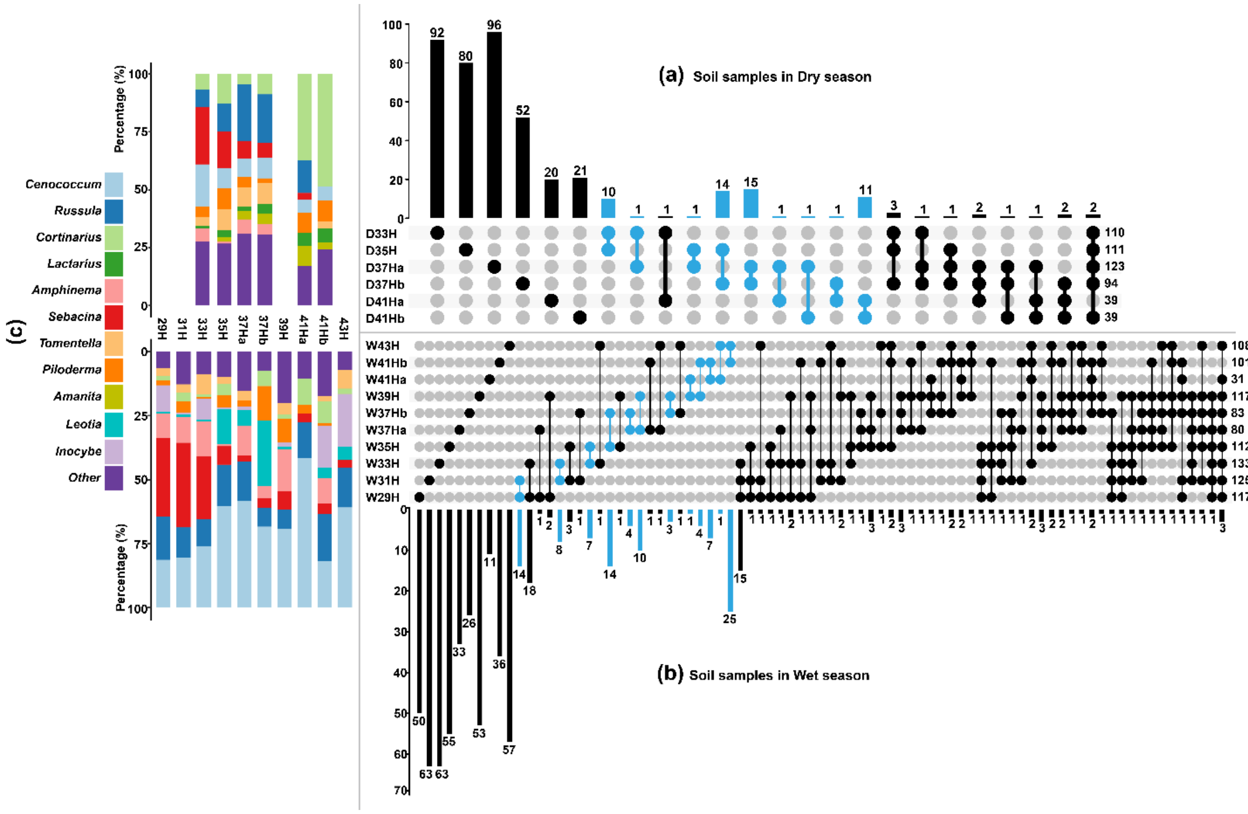

Fig. S6 Tableau stacked bar charts based on ASVs (relative abundance > 0.1%) from root samples (a) and soil samples (b) in dry and wet season. The number in brackets is the total number of ASVs (relative abundance > 0.1%) in each season.

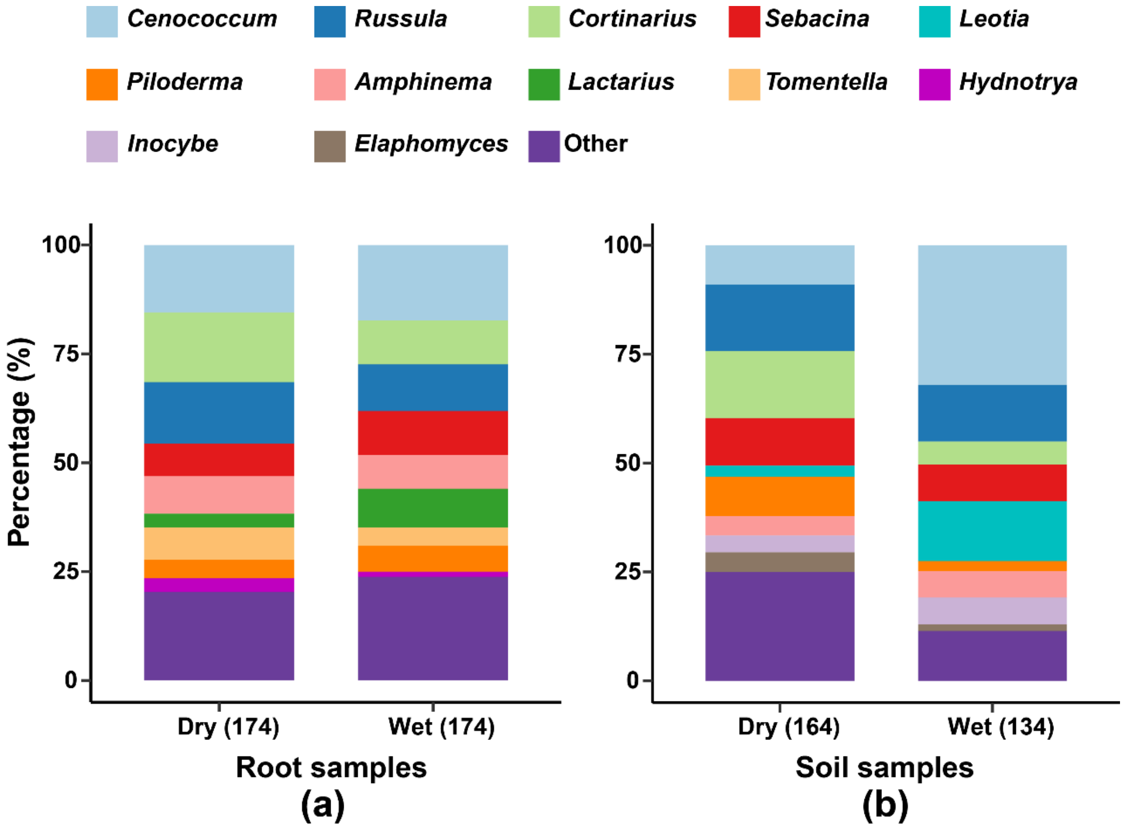

152 **Fig. S7** Venn diagrams of variation-partitioning results to show pure and shared contributions of slow-changing and fast-changing environmental variables to elevation and season variations of EcM fungal communities. The numbers in the graphs are the percentage of variance explained by the corresponding environmental factors. Slow, slow-changing environmental variables were pH, TP, and TK. Fast, fast-changing environmental variables were sea.MT, AN, AP, and AK. Other in each graph refers to other environment variables not shown in that graph.

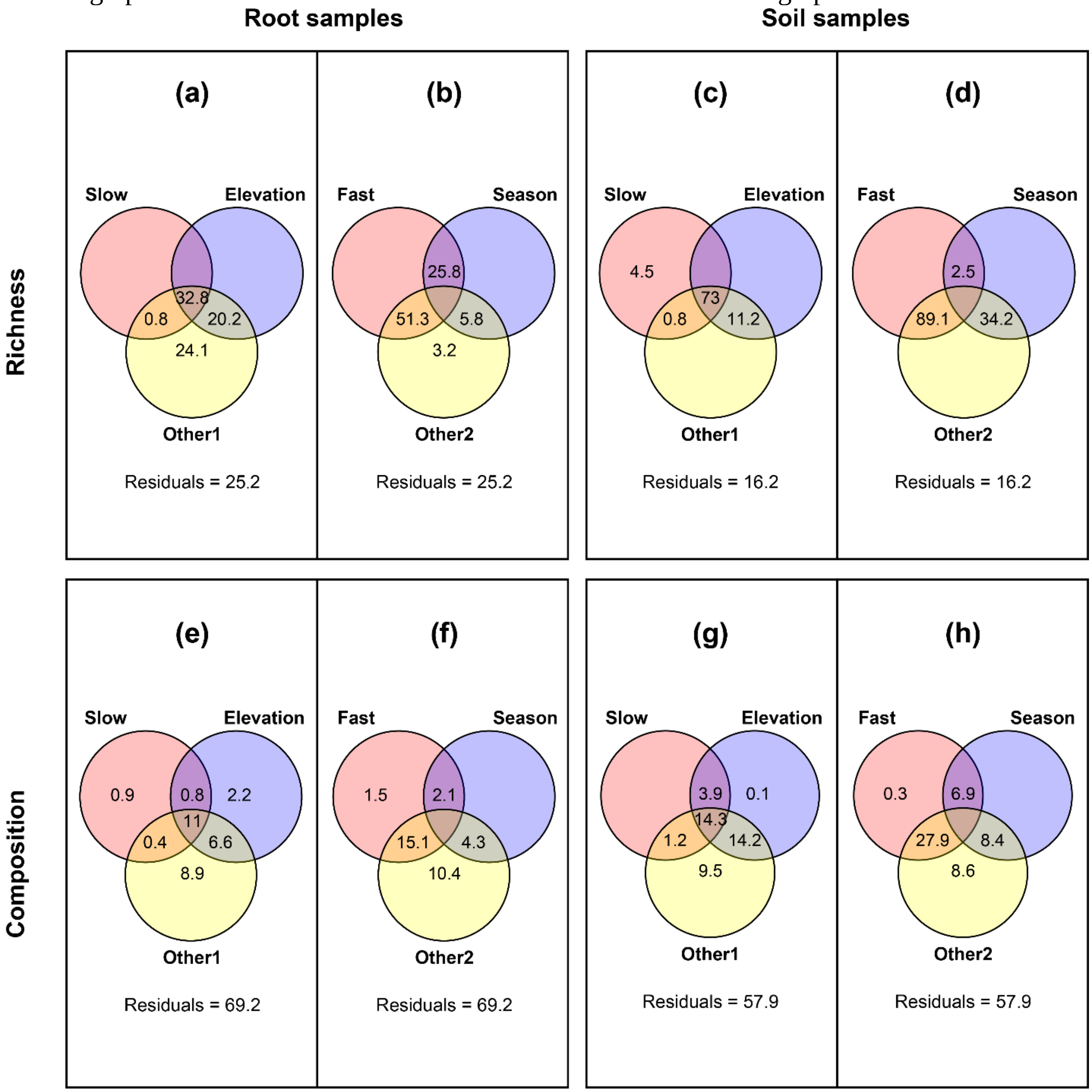

158

Table S1 Sampling plots and their corresponding environmental variables.

Plot code	Source	Elevation (m)	Season	Zone code	Date	Longitude E (°)	Latitude N (°)	em.GR	em.FR	em.abun	AMT (℃)
D33H1	SR	3292	Dry	D33H	2017.11.4	99.19468	28.28871	2	2	44	9.07
D33H2	SR	3289	Dry	D33H	2017.11.4	99.19519	28.28963	2	2	23	9.1
D35H1	SR	3571	Dry	D35H	2017.11.3	99.15615	28.31057	2	2	18	6.65
D35H2	SR	3549	Dry	D35H	2017.11.3	99.15711	28.31018	1	1	12	6.84
D35H3	SR	3574	Dry	D35H	2017.11.3	99.15854	28.30907	1	1	14	6.62
D37H1	SR	3699	Dry	D37Ha	2017.11.1	99.15004	28.30836	2	1	34	5.54
D37H2	SR	3807	Dry	D37Hb	2017.11.1	99.14616	28.31109	3	2	42	4.6
D37H3	SR	3775	Dry	D37Hb	2017.11.1	99.14741	28.30998	3	2	33	4.88
D37H4	SR	3736	Dry	D37Ha	2017.11.3	99.14976	28.30965	2	1	24	5.22
D37H5	SR	3710	Dry	D37Ha	2017.11.3	99.14997	28.30909	2	1	18	5.44
D41H1	SR	4161	Dry	D41Ha	2017.11.2	99.09669	28.32118	1	1	36	1.52
D41H2	SR	4229	Dry	D41Ha	2017.11.2	99.09536	28.32338	1	1	53	0.93
D41H3	SR	4193	Dry	D41Ha	2017.11.2	99.09155	28.32433	1	1	45	1.24
D41H4	SR	4226	Dry	D41Hb	2017.11.2	99.0868	28.32827	1	1	10	0.96
D41H5	SR	4231	Dry	D41Hb	2017.11.2	99.08631	28.32885	2	1	20	0.91
W29H1	S	2976	Wet	W29H	2018.8.20	99.18986	28.2683	1	1	67	11.82
W29H2	S	2987	Wet	W29H	2018.8.20	99.18825	28.26921	1	1	92	11.73
W29H3	S	2990	Wet	W29H	2018.8.20	99.18681	28.27007	2	2	190	11.7
W31H1	S	3099	Wet	W31H	2018.8.19	99.16276	28.29541	3	2	25	10.75
W31H2	S	3054	Wet	W31H	2018.8.19	99.16592	28.29312	2	2	94	11.14
W31H3	S	3104	Wet	W31H	2018.8.19	99.16893	28.29429	2	2	24	10.71
W33H1	SR	3309	Wet	W33H	2018.8.18	99.19417	28.28843	2	2	46	8.93
W33H2	SR	3296	Wet	W33H	2018.8.18	99.19526	28.29068	2	2	27	9.04
W33H3	S	3367	Wet	W33H	2018.8.18	99.19848	28.29042	1	1	78	8.42
W33H4	SR	3310	Wet	W33H	2018.8.19	99.16249	28.30028	2	2	84	8.92
W35H1	SR	3587	Wet	W35H	2018.8.17	99.155	28.31153	2	2	14	6.51
W35H2	SR	3597	Wet	W35H	2018.8.17	99.15655	28.31025	1	1	9	6.42
W35H3	SR	3572	Wet	W35H	2018.8.17	99.15842	28.30885	2	2	10	6.64

W35H4	S	3576	Wet	W35H	2018.8.17	99.15988	28.30655	3	2	18	6.61
W37H1	SR	3693	Wet	W37Ha	2018.8.12	99.14959	28.30932	2	1	48	5.59
W37H2	SR	3669	Wet	W37Ha	2018.8.12	99.14892	28.31114	2	1	45	5.8
W37H3	SR	3654	Wet	W37Ha	2018.8.12	99.14987	28.31192	3	2	36	5.93
W37H4	SR	3765	Wet	W37Hb	2018.8.13	99.14772	28.31022	3	3	40	4.96
W37H5	SR	3667	Wet	W37Hb	2018.8.14	99.14853	28.30928	3	2	36	5.81
W37H6	SR	3679	Wet	W37Hb	2018.8.14	99.14903	28.30856	3	2	51	5.71
W39H1	SR	3920	Wet	W39H	2018.8.14	99.13004	28.31909	2	1	32	3.62
W39H2	SR	3873	Wet	W39H	2018.8.15	99.12568	28.31951	3	2	18	4.02
W39H3	S	3882	Wet	W39H	2018.8.15	99.12575	28.32059	1	1	20	3.95
W39H4	SR	3975	Wet	W39H	2018.8.15	99.12334	28.32003	2	1	19	3.14
W39H5	S	3893	Wet	W39H	2018.8.15	99.12033	28.31987	2	1	23	3.85
W41H1	SR	4134	Wet	W41Ha	2018.8.11	99.09642	28.32181	1	1	31	1.76
W41H2	SR	4176	Wet	W41Ha	2018.8.11	99.09485	28.32264	1	1	40	1.39
W41H3	SR	4130	Wet	W41Ha	2018.8.11	99.09408	28.32434	1	1	34	1.79
W41H4	S	4139	Wet	W41Ha	2018.8.11	99.09641	28.32	1	1	28	1.71
W41H5	SR	4227	Wet	W41Hb	2018.8.16	99.08396	28.33157	2	1	8	0.95
W41H6	SR	4205	Wet	W41Hb	2018.8.16	99.08616	28.32981	2	1	14	1.14
W41H7	SR	4273	Wet	W41Hb	2018.8.16	99.06542	28.34267	2	1	26	0.55
W41H8	S	4092	Wet	W41Hb	2018.8.16	99.03414	28.35286	2	1	29	2.12
W43H1	SR	4510	Wet	W43H	2018.8.20	99.0011	28.37284	4	3	2133	-1.51
W43H2	S	4496	Wet	W43H	2018.8.20	99.00271	28.37241	4	3	1887	-1.39
W43H3	SR	4430	Wet	W43H	2018.8.21	99.00773	28.38785	4	3	3066	-0.82
W43H4	SR	4408	Wet	W43H	2018.8.21	99.00938	28.38641	4	3	4161	-0.63

Plot code	DMT (℃)	WMT (℃)	pH	OM (g kg^{-1})	TN (g kg^{-1})	TP (g kg^{-1})	TK (g kg^{-1})	AN (mg kg^{-1})	AP (mg kg^{-1})	AK (mg kg^{-1})
D33H1	4.52	NA	5.98	183.84	3.25	0.7	10	229.39	27.69	330.85
D33H2	4.55	NA	5.66	130.23	3.77	0.62	12.99	246.59	24.88	341.49
D35H1	2.07	NA	4.71	470.63	11.6	0.79	8.4	689.12	21.27	402.66
D35H2	2.27	NA	5.12	550.09	17.02	0.93	6.22	929.02	34.92	463.83

D35H3	2.05	NA	4.8	390.93	11.41	0.86	7.37	644.2	17.26	333.51
D37H1	0.95	NA	4.93	272.42	7.57	0.98	9.04	514.21	11.64	189.89
D37H2	0	NA	4.64	238.76	8.45	1.24	13.18	592.58	6.02	304.26
D37H3	0.28	NA	4.86	302.4	9.69	1.43	11.22	636.55	8.03	423.94
D37H4	0.62	NA	4.74	244.48	8.59	1.01	14.15	555.31	6.82	173.94
D37H5	0.85	NA	5.32	190.7	6.38	0.96	10.18	509.43	14.45	269.68
D41H1	-3.11	NA	3.9	388.28	7.07	0.61	13.78	454.95	9.23	354.79
D41H2	-3.71	NA	4.02	278.48	5.77	0.45	16.78	353.64	7.63	248.4
D41H3	-3.39	NA	3.99	346.72	8.19	0.67	12.68	465.46	12.84	248.4
D41H4	-3.68	NA	4.06	267.97	6.09	0.42	19.31	380.4	17.66	184.57
D41H5	-3.73	NA	3.99	355.22	6.29	0.37	17.38	422.45	22.08	269.68
W29H1	NA	16.35	6.15	85.48	2.42	0.78	22.51	178.5	28.23	239.51
W29H2	NA	16.25	6.35	84.45	2.1	0.85	24.15	153.55	37.54	181.79
W29H3	NA	16.23	5.49	38.49	0.94	0.52	31.36	79.66	16.34	158.18
W31H1	NA	15.29	5.75	217.96	4.58	0.9	15.73	155.47	75.48	331.32
W31H2	NA	15.68	6.34	123.78	3.14	0.48	17.2	221.69	12.98	218.52
W31H3	NA	15.25	6.17	51.57	1.31	0.32	16.96	95.01	8.82	145.07
W33H1	NA	13.48	6.27	122.79	2.1	0.52	9.26	149.71	15.75	260.49
W33H2	NA	13.59	6.9	166.46	4.56	0.76	10.32	278.31	25.06	491.34
W33H3	NA	12.98	6.27	59.1	1.29	0.52	14.78	97.89	13.97	131.95
W33H4	NA	13.47	5.38	118.84	1.67	0.73	14.85	119	28.63	173.92
W35H1	NA	11.09	4.78	577.13	14.88	1.05	8.64	458.74	53.19	530.69
W35H2	NA	11.01	5.33	448.74	11.28	0.69	10.41	357.01	33.38	402.15
W35H3	NA	11.22	4.77	409.67	9.85	0.8	7.27	298.47	27.83	365.42
W35H4	NA	11.19	4.41	613.68	11.12	1.1	6.56	333.98	34.57	541.19
W37H1	NA	10.18	4.28	288.57	7.29	0.92	7.79	252.4	40.51	158.18
W37H2	NA	10.39	4.44	237.82	7.12	1.26	15.36	284.07	41.7	168.68
W37H3	NA	10.51	4.66	184.19	6	0.91	11.3	224.57	24.66	166.05
W37H4	NA	9.56	4.57	243.68	8.3	1.14	23.23	271.6	37.74	223.77
W37H5	NA	10.4	4.88	244.98	8.4	1.22	9.46	350.29	40.11	273.61
W37H6	NA	10.3	4.32	257.87	7.29	0.94	8.11	254.32	43.68	234.26

W39H1	NA	8.23	4.47	280.85	7.6	0.96	10.55	284.07	29.02	210.65
W39H2	NA	8.63	4.69	363.02	9.1	1.11	5.01	301.35	31.4	278.86
W39H3	NA	8.55	4.94	325.41	8.75	1.21	6.77	322.46	25.26	250
W39H4	NA	7.75	5.36	197.87	5.5	0.98	6.34	223.61	13.97	339.19
W39H5	NA	8.46	4.78	320.79	8.58	1.01	5.11	282.15	33.18	260.49
W41H1	NA	6.39	3.94	296.8	5.16	0.46	17.5	181.86	25.65	223.77
W41H2	NA	6.02	3.98	292.17	6.3	0.55	17.25	201.54	31	234.26
W41H3	NA	6.42	3.92	405.31	7.55	0.55	12.1	199.62	37.34	276.23
W41H4	NA	6.34	3.9	467.15	7.99	0.56	10.93	262.96	39.72	386.41
W41H5	NA	5.59	3.63	305.37	6.37	0.6	17.86	214.01	30.8	208.03
W41H6	NA	5.77	3.89	288.24	6.19	0.49	17.66	200.58	37.34	250
W41H7	NA	5.19	5.27	130.2	4	0.91	18.25	170.83	12.58	229.01
W41H8	NA	6.75	4.04	157	3.56	0.54	21.53	135.32	55.76	116.21
W43H1	NA	3.15	4.59	305.37	10.32	1.04	14.4	358.93	25.65	210.65
W43H2	NA	3.27	4.55	290.73	7.83	0.76	14.35	273.51	22.68	160.81
W43H3	NA	3.84	4.89	188.54	7.89	1.32	17.63	319.58	23.47	396.9
W43H4	NA	4.03	4.83	255.97	10.26	1.34	13.78	367.57	30.8	328.7

Note: SR, both soil and root samples were collected; S, only soil samples were collected; em.GR, richness of EcM plant at genus level; em.FR, richness of EcM plant at family level; em.abun, the number of individuals of each EcM genus; AMT, annual mean temperature. DMT, dry-season mean temperature. WMT, wet-season mean temperature. OM, soil organic material. TN, total nitrogen. TP, total phosphorus. TK, total potassium. AN, alkaline-hydrolysable nitrogen. AP, available phosphorus. AK, available potassium. NA, not available.

Table S2 General descriptions of EcM fungi in different classification levels on Baima Snow Mountain.

Phylum	Phylogenetic lineage	Genus	Number of ASVs in each genus	Relative abundance (%)
Basidiomycota	/russula-lactarius	*Russula*	389	11
Basidiomycota	/cortinarius	*Cortinarius*	379	11.6
Basidiomycota	/tomentella-thelephora	*Tomentella*	368	3.7
Basidiomycota	/sebacina	*Sebacina*	246	5.4
Basidiomycota	/russula-lactarius	*Lactarius*	232	2.9
Basidiomycota	/amphinema-tylospora	*Amphinema*	164	8.3
Basidiomycota	/piloderma	*Piloderma*	143	4.3
Basidiomycota	/inocybe	*Inocybe*	108	1.7
Basidiomycota	/suillus-rhizopogon	*Suillus*	61	0.4
Basidiomycota	/amanita	*Amanita*	38	0.5
Basidiomycota	/laccaria	*Laccaria*	34	1
Basidiomycota	/tomentella-thelephora	*Thelephora*	30	0.3
Basidiomycota	/ramaria-gautieria	*Gautieria*	27	0.1
Basidiomycota	/boletus	*Imleria*	27	0.7
Basidiomycota	/boletus	*Boletus*	24	0.2
Basidiomycota	/hygrophorus	*Hygrophorus*	21	0.6
Basidiomycota	/tricholoma	*Tricholoma*	21	0.5
Basidiomycota	/tomentellopsis	*Tomentellopsis*	19	0.2
Basidiomycota	/pseudotomentella	*Pseudotomentella*	18	<0.1
Basidiomycota	/suillus-rhizopogon	*Rhizopogon*	16	0.3
Basidiomycota	/clavulina	*Clavulina*	12	0.3
Basidiomycota	/cantharellus	*Sistotrema*	12	0.1
Basidiomycota	/suillus-rhizopogon	*Truncocolumella*	10	<0.1
Basidiomycota	/amphinema-tylospora	*Tylospora*	10	0.5
Basidiomycota	/phellodon-bankera	*Phellodon*	9	<0.1
Basidiomycota	/hydnellum-sarcodon	*Sarcodon*	8	<0.1
Basidiomycota	/suillus-rhizopogon	*Chroogomphus*	7	<0.1

Basidiomycota	/ramaria-gautieria	*Gomphus*	7	<0.1
Basidiomycota	/hebeloma-alnicola	*Hebeloma*	7	0.1
Basidiomycota	/hydnellum-sarcodon	*Hydnellum*	7	0.1
Basidiomycota	/boletus	*Zangia*	7	0.1
Basidiomycota	/russula-lactarius	*Gymnomyces*	6	0.2
Basidiomycota	/boletus	*Leccinum*	5	<0.1
Basidiomycota	/albatrellus	*Leucogaster*	5	0.1
Basidiomycota	/clavulina	*Membranomyces*	5	2.5
Basidiomycota	/boletus	*Butyriboletus*	4	0.1
Basidiomycota	/cantharellus	*Hydnum*	4	<0.1
Basidiomycota	/hysterangium	*Hysterangium*	4	0.9
Basidiomycota	/austropaxillus	*Paxillus*	4	0.1
Basidiomycota	/albatrellus	*Albatrellus*	3	0.1
Basidiomycota	/byssocorticium	*Byssocorticium*	3	<0.1
Basidiomycota	/cantharellus	*Craterellus*	3	<0.1
Basidiomycota	/hebeloma-alnicola	*Hymenogaster*	3	<0.1
Basidiomycota	/boletus	*Xerocomus*	3	<0.1
Basidiomycota	/boletus	*Chamonixia*	2	<0.1
Basidiomycota	/russula-lactarius	*Lactifluus*	2	<0.1
Basidiomycota	/paralyophyllum	*Lyophyllum*	2	<0.1
Basidiomycota	/russula-lactarius	*Macowanites*	2	0.1
Basidiomycota	/boletus	*Phylloporus*	2	<0.1
Basidiomycota	/ramaria-gautieria	*Ramaria*	2	<0.1
Basidiomycota	/boletus	*Aureoboletus*	1	<0.1
Basidiomycota	/boletus	*Pulveroboletus*	1	<0.1
Ascomycota	/cenococcum	*Cenococcum*	408	30.3
Ascomycota	/tuber-helvella	*Helvella*	45	1.6
Ascomycota	/leotia	*Leotia*	34	1.4
Ascomycota	/hydnotrya	*Hydnotrya*	27	1.5
Ascomycota	/elaphomyces	*Elaphomyces*	21	0.7
Ascomycota	/tuber-helvella	*Tuber*	21	0.8
Ascomycota	/genea-humaria	*Humaria*	16	0.2

Ascomycota	/otidea	*Otidea*	10	0.3
Ascomycota	/geopora	*Geopora*	4	<0.1
Ascomycota	/terfezia-peziza depressa	*Peziza*	4	<0.1
Ascomycota	/marcelleina-peziza gerardii	*Hydnobolites*	3	<0.1
Ascomycota	/phaeohelotium	*Phaeohelotium*	1	<0.1
Ascomycota	/tarzetta	*Tarzetta*	1	<0.1
Ascomycota	/wilcoxina	*Trichophaea*	1	<0.1

Table S3 Significant effect of seasons, elevation zones and host genera identity on EcM fungal community composition detected by PERMANOVA.

**Root samples**								
**Zone**			**Host**			**Season**		
	R^2	Padj		R^2	Padj		R^2	Padj
overall test	0.19	0.001*	overall test	0.17	0.001*	overall test	0.03	0.001*
paired test			paired test			paired test		
33H/35H	0.06	0.001*	*Pinus*/*Quercus*	0.07	0.001*	D33H/W33H	0.11	0.001*
33H/37Ha	0.11	0.001*	*Pinus*/*Picea*	0.11	0.001*	D35H/W35H	0.14	0.001*
33H/37Hb	0.09	0.001*	*Pinus*/*Abies*	0.11	0.001*	D37Ha/W37Ha	0.09	0.013*
33H/41Ha	0.18	0.001*	*Pinus*/*Larix*	0.11	0.001*	D37Hb/W37Hb	0.09	0.003*
33H/41Hb	0.11	0.001*	*Pinus*/*Polygonum*	0.14	0.001*	D41Ha/W41Ha	0.08	0.052
35H/37Ha	0.08	0.001*	*Quercus*/*Picea*	0.06	0.007*	D41Hb/W41Hb	0.07	0.097
35H/37Hb	0.06	0.001*	*Quercus*/*Abies*	0.08	0.001*			
35H/41Ha	0.19	0.001*	*Quercus*/*Larix*	0.11	0.001*			
35H/41Hb	0.12	0.001*	*Quercus*/*Polygonum*	0.14	0.001*			
37Ha/37Hb	0.05	0.002*	*Picea*/*Abies*	0.06	0.011*			
37Ha/41Ha	0.23	0.001*	*Picea*/*Larix*	0.12	0.001*			
37Ha/41Hb	0.15	0.001*	*Picea*/*Polygonum*	0.16	0.001*			
37Hb/41Ha	0.19	0.001*	*Abies*/*Larix*	0.06	0.009*			
37Hb/41Hb	0.12	0.001*	*Abies*/*Polygonum*	0.14	0.001*			
41Ha/41Hb	0.1	0.001*	*Larix*/*Polygonum*	0.12	0.001*			
**Soil samples**								
**Zone**			**Season**					
	R2	Padj		R2	Padj			
overall test	0.43	0.001*	overall test	0.07	0.016*			
paired test			paired test					
33H/35H	0.25	0.01*	D33H/W33H	0.32	0.667			
33H/37Ha	0.29	0.012*	D35H/W35H	0.36	0.1			
33H/37Hb	0.3	0.038*	D37Ha/W37Ha	0.34	0.1			

33H/41Ha	0.43	0.012*	D37Hb/W37Hb	0.44	0.333				
35H/37Ha	0.27	0.01*	D41Ha/W41Ha	0.44	0.1				
35H/37Hb	0.19	0.031*	D41Hb/W41Hb	0.71	0.333				
35H/41Ha	0.43	0.012*							
37Ha/37Hb	0.16	0.059							
37Ha/41Ha	0.39	0.013*							
37Hb/41Ha	0.39	0.012*							

Note: *, significance at the level of $p < 0.05$
